# Supplementary material for: Spin-relaxation time in materials with broken inversion symmetry and large spin-orbit coupling
Source: Sci Rep. 2017 Aug 30;7:9949. doi: 10.1038/s41598-017-09759-0 (PMC5577210; doi:10.1038/s41598-017-09759-0)
Supplement: Supplementary file 1 — Supplementary Material [file 41598_2017_9759_MOESM1_ESM.pdf]

Supplementary information
Spin-relaxation time in materials with broken inversion symmetry and large spin-orbit coupling

Lénard Szolnoki,[1] Annamária Kiss,[2,3] Balázs Dóra,[4] and Ferenc Simon[1,*]

[1]*Department of Physics, Budapest University of Technology and Economics and MTA-BME Lendület Spintronics Research Group (PROSPIN), POBox 91, H-1521 Budapest, Hungary*
[2]*Institute for Solid State Physics and Optics, Wigner Research Centre for Physics, Hungarian Academy of Sciences, POBox 49, H-1525 Budapest, Hungary*
[3]*BME-MTA Exotic Quantum Phases Research Group, Budapest University of Technology and Economics, Budapest, Hungary*
[4]*Department of Theoretical Physics, Budapest University of Technology and Economics and MTA-BME Lendület Spintronics Research Group (PROSPIN), POBox 91, H-1521 Budapest, Hungary*
(Dated: June 23, 2017)

This Supplementary Material is organized as follows: we discuss the details of the $\mathcal{O}\left(\mathcal{L}/E_{\mathrm{F}}\right)$ approximation, which we employed in the main text. We further discuss the relationship between $\mathcal{S}$ and the dynamic spin-susceptibility and we also give an analytic treatment of the spin-dynamics in the clean limit. We present the equivalence of the *two-site motional narrowing NMR problem* and the 2D Bychkov-Rashba induced spin-relaxation for spins aligned perpendicular to the plane. The spin-relaxation is discussed when both Dresselhaus and Bychkov-Rashba spin-orbit couplings are present.

PACS numbers: 76.30.Pk, 71.70.Ej, 75.76.+j

I. THE EFFECT OF THE $\mathcal{O}\left(\mathcal{L}/E_{\mathrm{F}}\right)$ APPROXIMATION

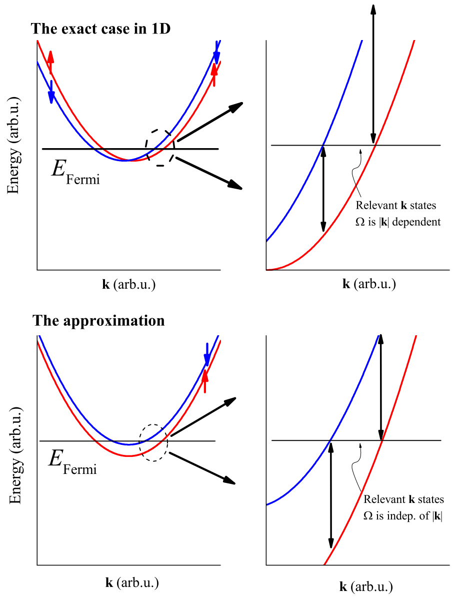

FIG. 1. Schematics of the $\mathcal{O}\left(\mathcal{L}/E_{\mathrm{F}}\right)$ approximation for a one-dimensional quadratic band dispersion. For such a dispersion, the Bychkov-Rashba type SOC shifts the up/down dispersions to the right/left, respectively. At the Fermi surface intersection, the SOC induced splitting is $|\mathbf{k}|$ dependent. In contrast, our approximation is equivalent to substituting it by a hypothetical, Zeeman-like split band structure, where the SOC induced splitting is independent of $|\mathbf{k}|$.

It was mentioned in the main text that both the Monte Carlo and the diagrammatic technique in Ref. 1 neglects the effect of the SOC on the Fermi surface. A proper calculation should consider that the bands are split due to the SOC and therefore the corresponding $\Omega(\mathbf{k})$ is $|\mathbf{k}|$ dependent due to this effect. In contrast, our approximation neglects this effect and Fig. 1 depicts this approximation for a one-dimensional band dispersion. The figure suggests that corrections to our approximation are on the order of $\mathcal{O}\left(\mathcal{L}/E_{\mathrm{F}}\right)$.

II. RELATIONSHIP BETWEEN $\mathcal{S}$ AND THE DYNAMIC SPIN-SUSCEPTIBILITY

We discussed in the main text that the time decay of a spin-polarized ensemble (described by $\underline{s}(t)$) is calculated with a Monte Carlo approach for both the clean and dirty cases. We found that the real part of its Fourier transform, Re$\underline{\underline{\mathcal{S}}}(\omega)$ can be conveniently displayed in order to demonstrate the spin-relaxation properties. Here, the i, i' components of $\underline{\underline{\mathcal{S}}}$ refer to the i' component of a spin ensemble which was originally polarized along i, with $i, i' = x, y,$ or z.

We also showed that a relation between Re$\underline{\underline{\mathcal{S}}}(\omega)$ and the dynamic spin-susceptibility, $\underline{\underline{\chi}}(\omega)$ holds:

$$\omega \cdot \mathrm{Re}\underline{\underline{\mathcal{S}}}(\omega) \propto \mathrm{Im}\underline{\underline{\chi}}(\omega). \tag{1}$$

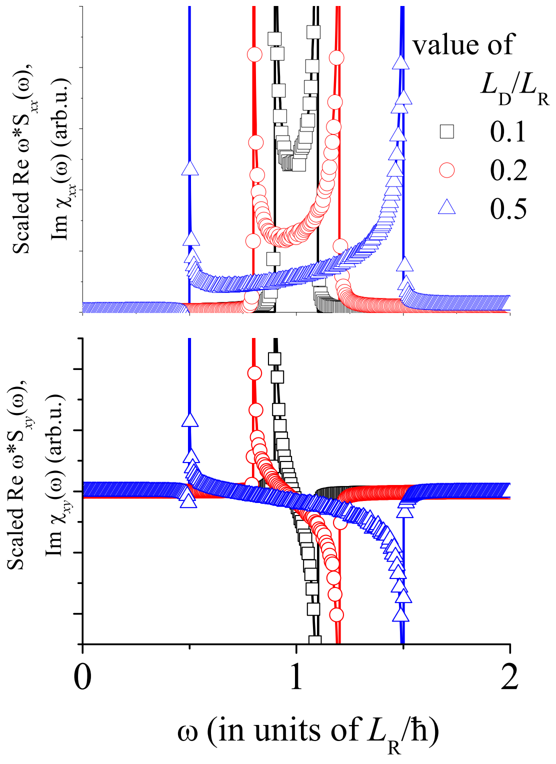

FIG. 2. Comparison of $\omega \cdot \mathcal{S}(\omega)$ as obtained from our Monte Carlo results (symbols) and the analytic calculations of Im$\underline{\underline{\chi}}(\omega)$ in Ref. 2 for various parameters of the SOC Hamiltonian and for various components of the $\underline{\underline{\chi}}$ and $\underline{\underline{\mathcal{S}}}$ tensors. Note the agreement between the two kinds of data besides a vertical scaling factor.

We present the above relationship on a quantitative agreement which we numerically obtained by comparing our Monte Carlo results on $\underline{\underline{\mathcal{S}(\omega)}}$ with analytic calculations of $\underline{\underline{\chi(\omega)}}$ for a particular Hamiltonian which is available in the literature (Ref. 2). The result is shown in Fig. 2 with the calculation details as follows.

Erlingsson *et. al.* (Ref. 2) calculated $\underline{\underline{\chi}}(\omega)$ for a two-dimensional electron gas for a Hamiltonian containing both Bychkov-Rashba and Dresselhaus type SOC terms as follows:

$$H_0 = \frac{\hbar^2 \boldsymbol{k}^2}{2m} + \frac{\mathcal{L}_\mathrm{R}}{k_\mathrm{F}}(s_x k_y - s_y k_x) + \frac{\mathcal{L}_\mathrm{D}}{k_\mathrm{F}}(s_y k_y - s_x k_x). \tag{2}$$

where $\mathcal{L}_\mathrm{R}$ and $\mathcal{L}_\mathrm{D}$ are the strengths of the Bychkov-Rashba and the Dresselhaus type spin-orbit couplings, respectively.

The dynamic spin-susceptibility was calculated in the absence of momentum relaxation and when $\mathcal{L}_\mathrm{R} \sim \mathcal{L}_\mathrm{D} \ll E_\mathrm{F}$. This limit matches the above discussed $\mathcal{O}(\mathcal{L}/E_\mathrm{F})$ approximation. Eq. 15 in Ref. 2 gives the dynamic spin-susceptibility for the various components as:

$$\chi_{xx}(\omega) = \lim_{\eta\to 0+} \frac{m}{2\pi\hbar^2}\left(1+\frac{\hbar^2(\omega+\mathrm{i}\eta)^2}{\sqrt{(\mathcal{L}_\mathrm{R}+\mathcal{L}_\mathrm{D})^2-\hbar^2(\omega+\mathrm{i}\eta)^2}\sqrt{(\mathcal{L}_\mathrm{R}-\mathcal{L}_\mathrm{D})^2-\hbar^2(\omega+\mathrm{i}\eta)^2}}\right)$$
$$\chi_{xy}(\omega) = \lim_{\eta\to 0+} \frac{m}{2\pi\hbar^2}\frac{\mathcal{L}_\mathrm{R}^2+\mathcal{L}_\mathrm{D}^2}{2\mathcal{L}_\mathrm{R}\mathcal{L}_\mathrm{D}}\left(\frac{\mathcal{L}_\mathrm{R}^2+\mathcal{L}_\mathrm{D}^2-\frac{4\mathcal{L}_\mathrm{R}^2\mathcal{L}_\mathrm{D}^2}{\mathcal{L}_\mathrm{R}^2+\mathcal{L}_\mathrm{D}^2}-\hbar^2(\omega+\mathrm{i}\eta)^2}{\sqrt{(\mathcal{L}_\mathrm{R}+\mathcal{L}_\mathrm{D})^2-\hbar^2(\omega+\mathrm{i}\eta)^2}\sqrt{(\mathcal{L}_\mathrm{R}-\mathcal{L}_\mathrm{D})^2-\hbar^2(\omega+\mathrm{i}\eta)^2}}-1\right) \tag{3}$$

Fig. 2. demonstrates a good agreement between the two kinds of data which supports the statement in the main text concerning the connection between $\underline{\underline{\mathcal{S}}}$ and $\underline{\underline{\chi}}$.

III. ANALYTIC TREATMENT OF THE SPIN DYNAMICS BY THE TIME EVOLUTION IN THE CLEAN LIMIT

We give an analytic description of the time evolution of the magnetization of an electron ensemble subjected to internal SOC fields to verify further the Monte Carlo method applied in the main text since this description is equivalent to the numerical method in the clean limit. We consider the SOC in the form $H_0 = \frac{1}{2}\mathbf{\Omega}(\mathbf{k})\cdot\boldsymbol{\sigma}$, where $\boldsymbol{\sigma}$ is a vector composed by the Pauli matrices, and $\mathbf{\Omega}(\mathbf{k})$ is the $\mathbf{k}$-dependent internal SOC field.

The time evolution of the state of an electron under the SOC is determined by the time evolution operator $U(t) = \exp(-iH_0t/\hbar)$. Supposing that the electron is initially in spin-up state, i.e. its spin is polarized along the z direction,

$$|\psi(0)\rangle = |\uparrow\rangle = \frac{v_2^{(-)}|+\rangle - v_2^{(+)}|-\rangle}{v_2^{(-)}v_1^{(+)}+v_2^{(+)}v_1^{(-)}}, \tag{4}$$

its state ket at time t is obtained as

$$|\psi(t)\rangle = \frac{v_2^{(-)}\mathrm{e}^{-iE_+t/\hbar}|+\rangle - v_2^{(+)}\mathrm{e}^{-iE_-t/\hbar}|-\rangle}{v_2^{(-)}v_1^{(+)}+v_2^{(+)}v_1^{(-)}} \tag{5}$$

by applying the time evolution operator. Here, $|\pm\rangle = [v_1^{(\pm)}, v_2^{(\pm)}]$ and $E_\pm$ are the eigenkets and eigenenergies of the Hamiltonian H_0. Finally, the time development of the z component of the electron spin is obtained as

$$\begin{aligned} S_z(t,\mathbf{k}) &= \langle\psi(t)|\hat{S}_z|\psi(t)\rangle \\ &= \frac{\Omega_x(\mathbf{k})^2+\Omega_y(\mathbf{k})^2}{\Omega(\mathbf{k})^2}\cos(\Omega(\mathbf{k})t) + \frac{\Omega_z(\mathbf{k})^2}{\Omega(\mathbf{k})^2}, \end{aligned} \tag{6}$$

where $\Omega = \sqrt{\Omega_x^2+\Omega_y^2+\Omega_z^2}$. The quantity $S_z(t)$, i.e. the z component of a spin ensemble, calculated by Monte Carlo method in the main text is obtained as

$$S_z(t) = \int_{\mathrm{F.S}} d\mathbf{k} S_z(t,\mathbf{k}) \tag{7}$$

within this approach, i.e. by integration over $\mathbf{k}$ on the Fermi surface. Arbitrary $\mathbf{\Omega}(\mathbf{k})$ can be considered in the Hamiltonian H_0 such as the two-dimensional Bychkov-Rashba SOC or the three-dimensional Dresselhaus case discussed in the main text. We note that for a complicated distribution of the SOC fields the $\mathbf{k}$ integration in Eq. (7) might not be performed analytically. In such a case, the integration can be performed by choosing random $\mathbf{k}$ values on the Fermi surface.

When this calculation is performed according to Eq. (6) for different model Hamiltonians such as those given in the main text, we obtained numerically identical results (data not shown) as for the Monte Carlo, and the analytic result for the dynamic spin susceptibility given in Eq. (3) is reproduced as well.

From expression (6) it is obvious that in a two-dimensional case, i.e. when $\Omega_z = 0$:

$$S_z(t,\mathbf{k}) = \cos(\Omega(\mathbf{k})t). \tag{8}$$

By taking the Bychkov-Rashba SOC, $\mathbf{\Omega}(\mathbf{k}) = \frac{\mathcal{L}}{\hbar k_\mathrm{F}}[k_x, k_y, 0]$, $\Omega(\mathbf{k})$ becomes $\mathbf{k}$ independent as $\Omega(\mathbf{k}) = \mathcal{L}/\hbar = \Omega$, which means a single oscillating component in $S_z(t)$ as it is shown by dashed curve in Fig.2 of the main text, and two Dirac-delta peaks at $\pm\Omega$ in the real part of the Fourier transform $\mathcal{S}(\omega)$. In three-dimensional cases, there is always a t-independent non-zero term in

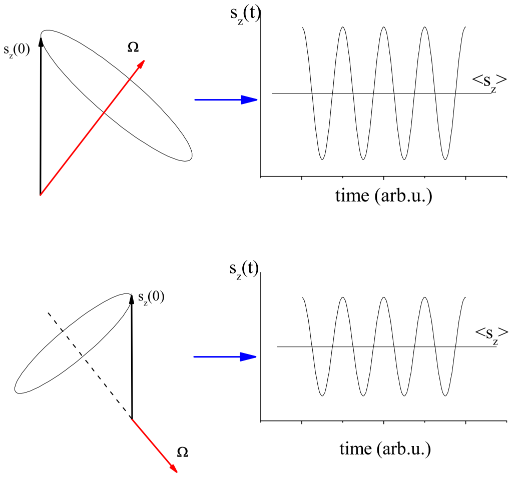

FIG. 3. Schematic depiction of the spin precession around the SOC fields and the corresponding $\mathbf{\Omega}(\mathbf{k})$ vectors when the spins start along the z direction at $t = 0$. Note that for an arbitrary $\mathbf{\Omega}(\mathbf{k})$ which is not in the $x - y$ plane, the precession retains a finite *positive* s_z value.

$S_z(t)$ coming from the last term in Eq. (6), which explains the finite S_z value in Fig.4 of the main text with $\Gamma = 0$. This non-zero and time independent term corresponds to a Dirac-delta function centered on $\omega = 0$ in $\mathcal{S}(\omega)$.

The origin of this effect is further supported by a geometric consideration which is depicted in Fig. 3. The presence of the Dirac-delta peak for $\mathcal{S}(\omega = 0)$ is a generic feature and its absence for the two-dimensional electron gas and the Bychkov-Rashba SOC is an exeption. For the latter, when the spins are aligned perpendicular to the 2D plane, all SOC fields are in the plane, i.e. the precession of the spins around the built in $\mathbf{\Omega}(\mathbf{k})$ results in a zero-averaged net magnetization. However, for a general distribution of the SOC fields and the corresponding $\mathbf{\Omega}(\mathbf{k})$ vectors, the precession of the spins retains a finite positive s_z component as Fig. 3. depicts. A straightforward geometric consideration shows that the $\langle s_z \rangle$, i.e. the Dirac delta function strength is given by Ω_z^2/Ω^2 (Ω_z and Ω are the z component and t he magnitude of the $\mathbf{\Omega}(\mathbf{k})$ vector, respectively) for a particular $\mathbf{\Omega}(\mathbf{k})$ component. Similarly, we obtain that the amplitude of the oscillation goes as $1 - \Omega_z^2/\Omega^2$, in full agreement with Eq. (6).

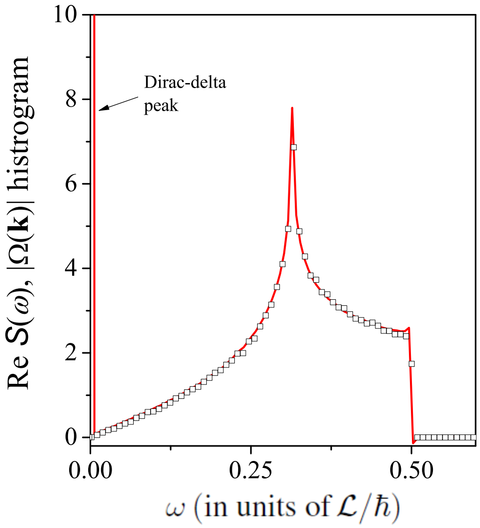

FIG. 4. Comparison of the Monte Carlo based Re$\mathcal{S}(\omega)$ (solid line) and the histogram of the $|\Omega(\mathbf{k})|$ (open symbols). Note that there is no scaling parameters between the two kinds of data.

In Fig. 4., we present the comparison between the Re$\mathcal{S}(\omega)$ and the histogram of the internal Larmor frequency distribution for the three-dimensional Dresselhaus model. The latter data integrated for the positive frequencies gives the average of the SOC

fields which coincides with the frequency value where it is peaked and its weighted integral, i.e the integral of the histogram values multiplied by the related frequency gives unity since all weights are summed up in this way.

IV. ANALOGY BETWEEN THE MOTIONAL NARROWING AND THE SPIN-RELAXATION FOR THE 2D BR MODELL

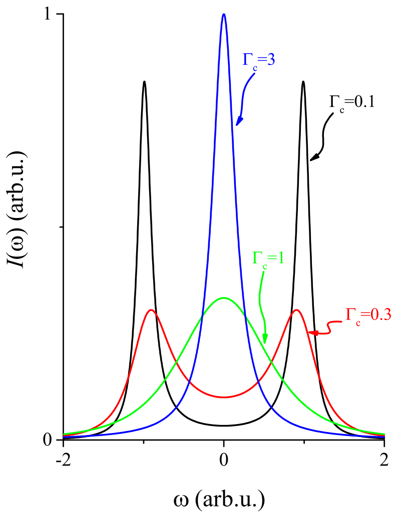

FIG. 5. Simulated lineshapes for the two-site NMR motional narrowing problem. Note that two peaks are observed for smaller values of Γ whose linewidth increases with increasing Γ. In contrast, a single, motionally narrowed peak is observed for larger Γ values, whose linewidth *decreases* with increasing Γ.

Abragam[3] considered the so-called two-site NMR motional narrowing problem: a nuclei is allowed to jump with the transition rate $\Gamma_{\rm c} = 1/\tau_{\rm c}$ between two sites with different local Larmor frequencies: $\pm\Omega$ around a central Larmor frequency (defined as zero in this case). The resulting NMR lineshape is shown in Fig. 5 for a fixed $\Omega = 1$ and different values of the jumping frequency, $\Gamma_{\rm c}$. The analogy between the spin-relaxation and the motional narrowing is clear: the $\pm\Omega$ local Larmor frequencies correspond to the built-in Zeeman field distribution of the spin-relaxation problem and the jumping frequency ($\Gamma_{\rm c}$) of the motional narrowing problem corresponds to the Γ momentum relaxation rate (besides a factor 2 which is discussed below). The analogy can be quantified for the simplest case as follows.

$$I(\omega) = \Re \frac{2{\rm i}\omega + 4\Gamma_{\rm c}}{(\Omega^2 - \omega^2) + 2{\rm i}\omega\Gamma_{\rm c}}. \tag{9}$$

The denominator of Eq. (9) has poles at $\omega_{1,2} = {\rm i}\left(\Gamma_{\rm c} \pm \sqrt{\Gamma_{\rm c}^2 - \Omega^2}\right)$, i.e. Eq. (9) can be rewritten as:

$$I(\omega) = \Re\left(\frac{A}{\omega - \omega_1} + \frac{B}{\omega - \omega_2}\right), \tag{10}$$

where $A = -{\rm i}\frac{\Gamma_{\rm c}+\Delta}{\Delta}$ and $B = {\rm i}\frac{\Gamma_{\rm c}-\Delta}{\Delta}$. Herein, we introduced $\Delta = \sqrt{\Gamma_{\rm c}^2 - \Omega^2}$. Evaluation of Eqs. (9) and (10) yields the curves shown in Fig. 5.

Our definition of Γ differs from that of $\Gamma_{\rm c}$ in Abragam's work in a factor 2 as discussed herein, as Γ corresponds to the momentum relaxation rate and Γ_c corresponds to the transition rate between the two states. The rate equations for the two state's populations:

$$
\begin{aligned}
\dot{n}_1 &= -\frac{1}{\tau_c}\left(n_1 - n_2\right) \\
\dot{n}_2 &= \frac{1}{\tau_c}\left(n_1 - n_2\right) \\
\partial_t\left(n_1 - n_2\right) &= -\frac{2}{\tau_c}\left(n_1 - n_2\right).
\end{aligned}
\tag{11}
$$

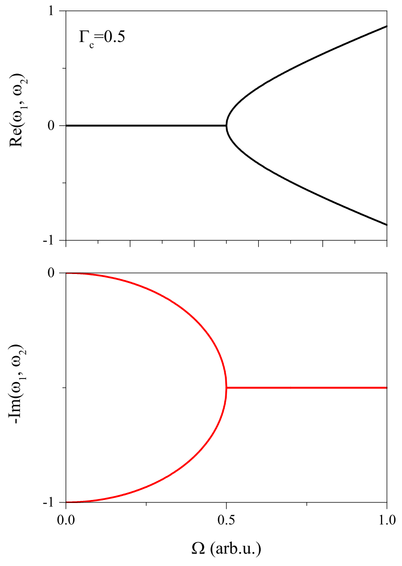

FIG. 6. Real and imaginary parts of the $\omega_{1,2}$ roots as defined above from the NMR motional narrowing problem.

We can see that $\frac{1}{\tau_{\mathrm{m}}} = \frac{2}{\tau_c}$, so $\Gamma = 2\Gamma_c$. With this change, the real and imaginary values of the above defined $\omega_{1,2}$ are shown in Fig. 6. The real part of the roots describe the position of the two peaks and the imaginary parts describe the linewidths in agreement with Fig. 6. Remarkably, this figure is *identical* to the spin-relaxation problem for the 2D Rashba model in the main paper with a straightforward identification of the correspondence of the two parameters.

V. SPIN-RELAXATION WHEN DRESSELHAUS AND BYCHKOV-RASHBA SPIN-ORBIT COUPLINGS ARE PRESENT

Both the Dresselhaus and Bychkov-Rashba spin-orbit couplings are present in III-V sermiconductor based quantum wells. The earlier is due to the bulk inversion symmetry breaking while the latter is due to a longitudinal electric field acting on the quantum well.

When the growth direction is along the $[0,0,1]$, both the Dresselhaus and the Bychkov-Rashba Hamiltonian has the usual form which is mentioned in the main text and the resulting the Larmor-frequency distribution reads:

$$
\boldsymbol{\Omega}(\boldsymbol{k}) = \frac{\mathcal{L}_{\mathrm{D}}}{\hbar k_{\mathrm{F}}^3}\begin{bmatrix} k_x\left(k_y^2 - k_z^2\right) \\ k_y\left(k_z^2 - k_x^2\right) \\ k_z\left(k_x^2 - k_y^2\right)\end{bmatrix} + \frac{\mathcal{L}_{\mathrm{R}}}{\hbar k_{\mathrm{F}}}\begin{bmatrix} -k_y \\ k_x \\ 0 \end{bmatrix},
\tag{12}
$$

When the growth direction is along the $[1,1,1]$, we take the z-axis along this direction, therefore the Bychkov-Rashba Hamiltonian is retained and the Dresselhaus one is transformed. The resulting Larmor-frequency distribution reads:

$$\boldsymbol{\Omega}(\boldsymbol{k}) = \frac{\mathcal{L}_{\mathrm{D}}}{2\sqrt{3}\hbar k_{\mathrm{F}}^{3}} \begin{bmatrix} -k_y(k_x^2+k_y^2)-(k_y^2-2k_xk_y-k_x^2)k_z+4k_yk_z^2 \\ k_x(k_x^2+k_y^2)+(k_x^2-2k_xk_y-k_y^2)k_z-4k_xk_z^2 \\ (k_x-k_y)(k_x^2+4k_xk_y+k_y^2) \end{bmatrix} + \frac{\mathcal{L}_{\mathrm{R}}}{\hbar k_{\mathrm{F}}} \begin{bmatrix} -k_y \\ k_x \\ 0 \end{bmatrix}. \tag{13}$$

In our examples we use $\mathcal{L}_{\mathrm{D}} = \mathcal{L}_{\mathrm{R}} = \mathcal{L}$.

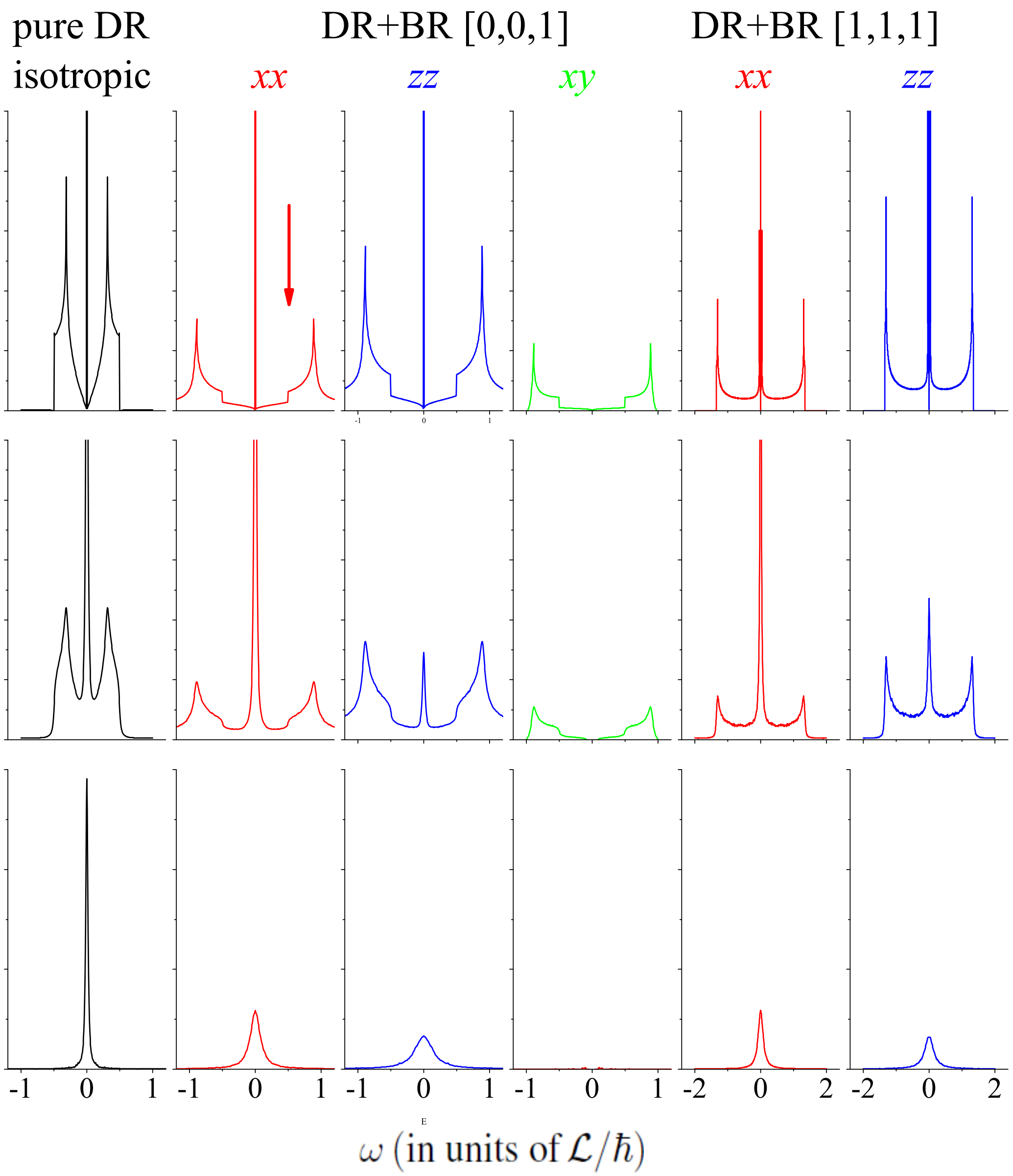

FIG. 7. The components of Re$\underline{\underline{\mathcal{S}}}(\omega)$ for the various SOC configurations and for different values of Γ. Γ from top to bottom is 0, $0.02\mathcal{L}$, and $5\mathcal{L}$. Note that the ω axis are scaled differently for the last two columns of figures.

In Fig. 7., we show the components of Re$\underline{\underline{\mathcal{S}}}(\omega)$ for the various SOC configurations and for different values of Γ. The first column is for the pure Dresselhaus SOC and is an identical result to that given in the main text. Γ values in the rows are 0, $0.02\mathcal{L}$, and $5\mathcal{L}$, respectively. The scales are the same for the first two rows of data but are different for the third one (with large Γ).

The important observations are: i) the overall SOC field becomes larger as expected with a singular (step-like) feature (indicated by an arrow in the figure), ii) the spin-relaxation becomes anisotropic when the Bychkov-Rashba term is also present and even an xy term is present for the $[0,0,1]$ growth direction, which however vanishes in the D'yakonov-Perel' regime, iii) the

TABLE I. The spin-relaxation broadening parameter, Γ_s in the D'yakonov-Perel' regime for the various SOC combinations in Fig. 7. for $\Gamma = 5\mathcal{L}$. The definition of Γ_s is given in the text

SOC model and direction	Γ_s in units of $\mathcal{L}$
pure Dresselhaus	0.017
Dresselhaus+Rashba $[0, 0, 1]$ xx direction	0.091
Dresselhaus+Rashba $[0, 0, 1]$ zz direction	0.161
Dresselhaus+Rashba $[1, 1, 1]$ xx direction	0.088
Dresselhaus+Rashba $[1, 1, 1]$ zz direction	0.161

anisotropy remains for the $[1, 1, 1]$ growth direction but the xy term is zero, iv) a single Lorentzian component is observed in the Dyakonov-Perel regime but with varying widths which are summarized in Table I. The broadening parameter, Γ_s is the HWFM of the Lorentzian curves in the figures.

These parameters indicate how strong the spin-relaxation in these models is. E.g. the 0.017 value is related to the corresponding spatial average of the $\mathcal{S}_{clean}$ function.

* Corresponding author: f.simon@eik.bme.hu

[1] Burkov, A. A. & Balents, L. Spin relaxation in a two-dimensional electron gas in a perpendicular magnetic field. *Phys. Rev. B* **69**, 245312 (2004).
[2] Erlingsson, S. I., Schliemann, J. & Loss, D. Spin susceptibilities, spin densities, and their connection to spin currents. *Phys. Rev. B* **71**, 035319 (2005).
[3] Abragam, A. *Principles of Nuclear Magnetism* (Oxford University Press, Oxford, England, 1961).
