# Supplementary material for: Spin-relaxation time in materials with broken inversion symmetry and large spin-orbit coupling
Source: Sci Rep. 2017 Aug 30;7:9949. doi: 10.1038/s41598-017-09759-0 (PMC5577210; doi:10.1038/s41598-017-09759-0)
Supplement: Supplementary file 2 — The Monte Carlo code of the calculations in C++ [file 41598_2017_9759_MOESM2_ESM.zip › DP_Monte_Carlo/doc/latex/refman.pdf]

Dyakonov Perel Monte Carlo simulation

Generated by Doxygen 1.8.13

Contents

1 Main Page **1**

2 Hierarchical Index **5**

2.1 Class Hierarchy 5

3 Class Index **7**

3.1 Class List 7

4 File Index **9**

4.1 File List 9

5 Class Documentation **11**

5.1 autocorr Class Reference 11

5.1.1 Detailed Description 12

5.1.2 Constructor & Destructor Documentation 12

5.1.2.1 autocorr() 12

5.1.3 Member Function Documentation 12

5.1.3.1 get_autocorr() 12

5.1.3.2 push() 12

5.2 buffer< T > Class Template Reference 13

5.2.1 Detailed Description 13

5.2.2 Constructor & Destructor Documentation 13

5.2.2.1 buffer() 13

5.2.3 Member Function Documentation 14

5.2.3.1 get_eff_size() 14

5.2.3.2 operator[]() 14
5.2.3.3 push() 14
5.3 randgen::gen Class Reference 15
5.3.1 Detailed Description 15
5.3.2 Member Function Documentation 15
5.3.2.1 getGen() 16
5.3.2.2 Instance() [1/2] 16
5.3.2.3 Instance() [2/2] 16
5.4 Progress Struct Reference 17
5.5 SingleSpin Class Reference 17
5.5.1 Detailed Description 18
5.5.2 Member Enumeration Documentation 18
5.5.2.1 meas_t 18
5.5.2.2 model_t 19
5.5.3 Constructor & Destructor Documentation 19
5.5.3.1 SingleSpin() 20
5.5.4 Member Function Documentation 20
5.5.4.1 FillSzVec() 20
5.5.4.2 GetFirstTime() 20
5.5.4.3 GetLastTime() 21
5.5.4.4 GetSpin() 21
5.5.4.5 Step() 21
5.6 SingleSpinAutocorr Class Reference 22
5.6.1 Detailed Description 22
5.6.2 Constructor & Destructor Documentation 23
5.6.2.1 SingleSpinAutocorr() 23
5.6.3 Member Function Documentation 23
5.6.3.1 GetAutocorr() 23
5.6.3.2 Step() 24

6 File Documentation **25**
6.1 include/la.h File Reference 25
6.1.1 Function Documentation 25
6.1.1.1 Rotate() 25

Index **27**

Chapter 1

Main Page

Compilation

The program is mainly developed on a Linux Debian distribution. Compilation depends on the *libarmadillo-dev* *libboost-program-options-dev* and *libboost-random-dev* packages. Running a single *make* command compiles the program to the path *bin/main*.

Usage

The program can be operated with command line options, it allows scripting large number of simulations with different parameters.

```
$ bin/main --help
Allowed options:
  -h [ --help ]                   produce help message
  -v [ --version ]                print version number
  --autocorr                      set autocorr measurement
  --spins arg (=10)               set number of spins
  --duration arg (=300)           set simulation duration
  --timestep arg (=1)             set timestep
  --omega arg (=0.20000000000000001) set the absolute value of Larmor
                                  precession
  --delta_omega arg (=0)          set the width of omega distribution
  --seed arg (=rand)              set the seed for the random generator
  -o [ --output ] arg (=-)        output file path
  -m [ --model ] arg (=naiv)      name of the model
  --meas arg (=prep)              name of measurement method
  -b [ --B_meas ] arg (=0)        measurement field
  --tmin arg (=0)                 starting time, B_meas turns in at t=0
```

As there are default arguments for all options (as seen in the help output), with no arguments the program outputs the result of a sample simulation. The output contains a header which contains the simulation parameters so the simulation can be reproduced. After the header it outputs the spin component of interest in the function of time. The time interval and sampling interval is the same as specified in the command line.

```
$ bin/main | head -20
# Djakonov-Perel simulation
# t=0 Sz=1, no magnetic field
#  version: commit_17cc1d6e5275608347fa311f7f7e64735470c311
#  spins: 10
#  duration: 300
#  timestep: 1
#  omega: 0.2
#  seed: 2494990189
```

```
#  model: naiv
#  meas: prep
#  B_meas: 0
#  tmin: 0
#  autocorr: false
# t, Sz
0, 1
1, 0.990826
2, 0.97055
3, 0.946542
4, 0.925218
5, 0.910056
```

Command line options

We can see from the output of "bin/main --help" that there are many options regarding to a single run of the simulation. Here I describe the details how these options work.

- **help** Outputs the brief description of command line options.
- **version** Outputs the version number. If the program is compiled in a clean state git repository then it is the commit hash of the given version.
- **duration** Sets the simulation time window's length.
- **timestep** Sets the interval between time samples.
- **tmin** Sets the starting time of the measurement, it is useful for measurements with step function external magnetic field, where it is turned on at t=0.
- **omega** The primary parameter of the underlying SOC model. It is typically the amplitude of the Larmor angular frequency due to the SOC field. The exact meaning depends on the model in question.
- **delta_omega** The secondary paramter for the SOC. It typically describes the deviation of amplitude of the Larmor angular frequency. The exact meaning is model dependent.
- **model** The name of the spin model selected. It also selects the initial condition and the spin direction of interest. The Hamiltonian has the form:

  $$H_{\mathrm{SOC}} = \hbar \boldsymbol{\Omega}(\boldsymbol{k}) \boldsymbol{s}$$

  In all models the exact $\boldsymbol{\Omega}(\boldsymbol{k})$ are paremetrized by two parameters, Ω and $\Delta\Omega$. These can be set by the options **omega** and **delta_omega** respectively.

  The available models:

  – *naiv* Fully isotropic 3D SOC model, we assume a spherical Fermi-surface.

  $$\boldsymbol{\Omega}(\boldsymbol{k}) = \Omega \frac{\boldsymbol{k}}{k_{\mathrm{F}}}$$

  – *burkov_2d* 2DEG model with Rashba SOC, z axis relaxation. The shape of the Fermi surface is a circle.

  $$\boldsymbol{\Omega}(\boldsymbol{k}) = \frac{\Omega}{k_{\mathrm{F}}} [-k_y, k_x, 0]$$

  – *burkov_2d_Sx* 2DEG model with Rashba SOC, x axis relaxation.

  – *burkov_2d_angle* 2DEG model with Rashba SOC, spins started polarized at a 45° angle to the z axis, z component gathered.

  – *burkov_2d_angle_sx* 2DEG model with Rashba SOC, spins started polarized at a 45° angle to the z axis, x component gathered.

  – *rashba_3d* 3D model with Rashba SOC. The SOC Hamiltonian is the same as in *burkov_2d*, but now we allow wave numbers in the z direction. We assume a spherical Fermi-surface.

– *mixed_3d* 3D model with both isotropic and Rashba SOC.

$$\mathbf{\Omega}(\boldsymbol{k}) = \Omega\frac{\boldsymbol{k}}{k_{\mathrm{F}}} + \frac{\Delta\Omega}{k_{\mathrm{F}}}[-k_y, k_x, 0]$$

– *mn_1d* 1D model which exactly gives back the result of motional narrowing. The "Fermi-surface" is two points $k = \pm k_{\mathrm{F}}$, $\mathbf{\Omega}(\boldsymbol{k}) = \Omega k/k_{\mathrm{F}}$. We start the spin from the z axis, the SOC field is perpendicular to the z axis.

– *dresselhaus* 3D model with dresselhaus SOC, z axis relaxation.

$$\mathbf{\Omega}(\boldsymbol{k}) = \frac{\Omega}{k_{\mathrm{F}}^3}\begin{bmatrix} k_x(k_y^2 - k_z^2) \\ k_y(k_z^2 - k_x^2) \\ k_z(k_x^2 - k_y^2) \end{bmatrix}$$

– *dresselhaus_xy* 3D model with dresselhaus SOC, spins started with x direction polarization, y component gathered.

– *rashba_dressel_2d_z* 2DEG model with both rashba and dresselhaus SOC, z axis relaxation.

$$\mathbf{\Omega}(\boldsymbol{k}) = \frac{\Omega}{k_{\mathrm{F}}}[-k_y, k_x, 0] + \frac{\Delta\Omega}{k_{\mathrm{F}}}[k_x, -k_y, 0]$$

– *rashba_dressel_2d_x* 2DEG model with both rashba and dresselhaus SOC, x axis relaxation.

– *rashba_dressel_2d_xy* 2DEG model with both rashba and dresselhaus SOC, spins started with x direction polarization, y component gathered.

– *rashba_dressel_3d_x* 3D model with both Dresselhaus and Rashba SOC, x axis relaxation. We assume [0,0,1] growth direction.

$$\mathbf{\Omega}(\boldsymbol{k}) = \frac{\Omega}{k_{\mathrm{F}}^3}\begin{bmatrix} k_x(k_y^2 - k_z^2) \\ k_y(k_z^2 - k_x^2) \\ k_z(k_x^2 - k_y^2) \end{bmatrix} + \frac{\Delta\Omega}{k_{\mathrm{F}}}\begin{bmatrix} -k_y \\ k_x \\ 0 \end{bmatrix}$$

– *rashba_dressel_3d_z* 3D model with both Dresselhaus and Rashba SOC, z axis relaxation.

– *rashba_dressel_3d_xz* 3D model with both Dresselhaus and Rashba SOC, spins started with x direction polarization, z component gathered.

– *rashba_dressel_3d_xy* 3D model with both Dresselhaus and Rashba SOC, spins started with y direction polarization, y component gathered.

– *rashba_dressel_3d_111_xx* 3D model with both Dresselhaus and Rashba SOC, [1,1,1] growth direction, x axis relaxation. The coordinate system's z axis is aligned to the growth direction.

$$\mathbf{\Omega}(\boldsymbol{k}) = \frac{\Omega}{2\sqrt{3}k_{\mathrm{F}}^3}\begin{bmatrix} -k_y(k_x^2 + k_y^2) - (k_y^2 - 2k_xk_y - k_x^2)k_z + 4k_yk_z^2 \\ k_x(k_x^2 + k_y^2) + (k_x^2 - 2k_xk_y - k_y^2)k_z - 4k_xk_z^2 \\ (k_x - k_y)(k_x^2 + 4k_xk_y + k_y^2) \end{bmatrix} + \frac{\Delta\Omega}{k_{\mathrm{F}}}\begin{bmatrix} -k_y \\ k_x \\ 0 \end{bmatrix}.$$

– *rashba_dressel_3d_111_zz* 3D model with both Dresselhaus and Rashba SOC, [1,1,1] growth direction, z axis relaxation.

- **autocorr**: If this option is set, then <s(t+tau)s(t)> time averaged autocorrelation is measured for a single spin. At this point the meaning of the options **spins** and **duration** changes. **duration** becomes the time window from where we take the tau values, **timestep** is the sampling interval for the **tau** values. The simulation is actually ran for **duration** × **spins** time, so the resulting graph has similar errors as an ensamble measurement.

- **seed** The random seed of the random generator. Potentially useful for debugging as a fix value guarantees deterministic runs.

- **output** The output file for the simulation, default is stdout.

Chapter 2

Hierarchical Index

2.1 Class Hierarchy

This inheritance list is sorted roughly, but not completely, alphabetically:

- buffer< T > 13
- buffer< double > 13
  - autocorr 11
- randgen::gen 15
- Progress 17
- SingleSpin 17
  - SingleSpinAutocorr 22

Chapter 3

Class Index

3.1 Class List

Here are the classes, structs, unions and interfaces with brief descriptions:

autocorr
Class for gathering autocorrelation of time series data 11
buffer< T >
A circular buffer template class 13
randgen::gen
Random generator singleton 15
Progress 17
SingleSpin
Spin relaxation experiment class mainly for ensamble measurements 17
SingleSpinAutocorr
Spin relaxation experiment class for autocorrelation measurements 22

Chapter 4

File Index

4.1 File List

Here is a list of all documented files with brief descriptions:

include/**autocorr.h** . . . . ??
include/**buffer.h** . . . . ??
include/la.h 25
include/**random.h** . . . . ??
include/**singlespin.h** . . . . ??

Chapter 5

Class Documentation

5.1 autocorr Class Reference

Class for gathering autocorrelation of time series data.

#include <autocorr.h>

Inheritance diagram for autocorr:

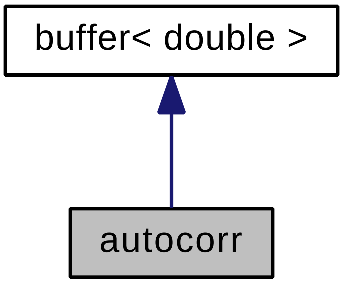

Collaboration diagram for autocorr:

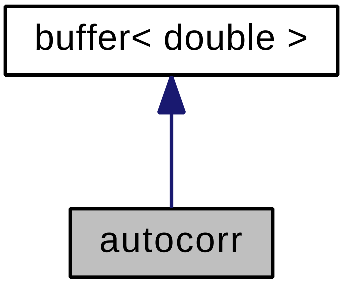

Public Member Functions

- autocorr (size_t size)

  Constructor.
- void push (const double &value)

  Pushes an element.
- std::unique_ptr< std::vector< double > > get_autocorr ()

  Gets the autocorrelation vector.

5.1.1 Detailed Description

Class for gathering autocorrelation of time series data.

For a time series a_0, a_1, a_2 ... it collects the mean values <a_i∗a_i>, <a_(i+1)∗a_i>, <a_(i+2)∗a_i> ...

5.1.2 Constructor & Destructor Documentation

5.1.2.1 autocorr()

```
autocorr::autocorr (
            size_t size )
```

Constructor.

Parameters

size	The allocated size of the buffer.

5.1.3 Member Function Documentation

5.1.3.1 get_autocorr()

```
std::unique_ptr< std::vector< double > > autocorr::get_autocorr ( )
```

Gets the autocorrelation vector.

Returns

An std::unique_ptr<std::vector<double>> containing the autocorrelation vector.

The elements of the vector are the index averaged mean values of <a_i∗a_i>, <a_(i+1)∗a_i>, <a_(i+2)∗a_i> ... Where a_i are the pushed inside the buffer.

5.1.3.2 push()

```
void autocorr::push (
            const double & value )  [virtual]
```

Pushes an element.

Parameters

value	The pushed element.

The pushed element will be available at object[get_eff_size()-1].

Reimplemented from buffer< double >.

The documentation for this class was generated from the following files:

- include/autocorr.h
- src/autocorr.cpp

5.2 buffer< T > Class Template Reference

A circular buffer template class.

```
#include <buffer.h>
```

Public Member Functions

- buffer (size_t size)

  Constructor.
- T & operator[ ] (size_t idx)

  Access specified element.
- virtual void push (const T &value)

  Pushes an element.
- size_t get_size ()

  Gets the allocated size of the buffer.
- size_t get_eff_size ()

  Gets the apparent size of the buffer.

5.2.1 Detailed Description

template<typename T>
class buffer< T >

A circular buffer template class.

This class acts like std::vector, except it "forgets" pushed elements that no longor fit inside.

5.2.2 Constructor & Destructor Documentation

5.2.2.1 buffer()

```
template<typename T >
buffer< T >::buffer (
              size_t size )
```

Constructor.

Parameters

size	The allocated size of the buffer.

5.2.3 Member Function Documentation

5.2.3.1 get_eff_size()

```
template<typename T>
size_t buffer< T >::get_eff_size ( )  [inline]
```

Gets the apparent size of the buffer.

The apparent size increases every time an element is pushed.

5.2.3.2 operator[]()

```
template<typename T >
T & buffer< T >::operator[ ] (
             size_t idx )
```

Access specified element.

Parameters

idx	Index of the given element.

Returns

The specified element.

Exceptions

std::out_of_range	

The maximal idx available increases for every push() operation. After the buffer gets full, then the minimal available index increases as well. If idx is not in legal range it throws std::out_of_range.

5.2.3.3 push()

```
template<typename T>
void buffer< T >::push (
             const T & value )  [virtual]
```

Pushes an element.

Parameters

value	The pushed element.

The pushed element will be available at object[get_eff_size()-1].

Reimplemented in autocorr.

The documentation for this class was generated from the following file:

- include/buffer.h

5.3 randgen::gen Class Reference

Random generator singleton.

`#include <random.h>`

Public Member Functions

- pseudogen & getGen ()

  Gets the underlying random generator.

Static Public Member Functions

- static gen ∗ Instance ()

  Gets the unique instance of the random generator.
- static gen ∗ Instance (uint32_t seed)

  Gets the unique instance of the random generator.

5.3.1 Detailed Description

Random generator singleton.

The random generator singleton used throughout the codebase. It prevents the creation of multiple independent generators, which could potentially hurt the randomness of the simulation.

5.3.2 Member Function Documentation

5.3.2.1 getGen()

```
pseudogen & randgen::gen::getGen ( )
```

Gets the underlying random generator.

Returns

The underlying random generator.

The returntype is pseudogen typedefd to boost::random::mt19937.

5.3.2.2 Instance() [1/2]

```
gen * randgen::gen::Instance ( )  [static]
```

Gets the unique instance of the random generator.

Returns

Pointer to the instance.

It constructs the generator with a random seed (using the OS's entropy pool) if it wasn't already constructed.

5.3.2.3 Instance() [2/2]

```
gen * randgen::gen::Instance (
            uint32_t seed )  [static]
```

Gets the unique instance of the random generator.

Parameters

seed	The seed passed to the constructor of the random generator.

Returns

Pointer to the instance.

The seed parameter is omitted if the instance already exists.

The documentation for this class was generated from the following files:

- include/random.h
- src/random.cpp

5.4 Progress Struct Reference

Public Member Functions

- void **MLSend** (MLINK lp)

Public Attributes

- std::string **Mathematica_variable**
- double **progress**

The documentation for this struct was generated from the following file:

- mathlink/mathlink.cpp

5.5 SingleSpin Class Reference

Spin relaxation experiment class mainly for ensamble measurements.

```
#include <singlespin.h>
```

Inheritance diagram for SingleSpin:

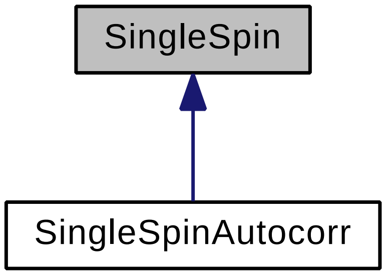

Public Types

- enum model_t {
naiv, burkov_2d, burkov_2d_Sx, burkov_2d_angle,
burkov_2d_angle_sx, rashba_3d, mixed_3d, mn_1d,
dresselhaus, dresselhaus_xy, rashba_dressel_2d_z, rashba_dressel_2d_x,
rashba_dressel_2d_xy, rashba_dressel_3d_x, rashba_dressel_3d_z, rashba_dressel_3d_xz,
rashba_dressel_3d_xy, rashba_dressel_3d_111_xx, rashba_dressel_3d_111_zz }

    An enum type for the underlying Hamiltonian and direction of interest.
- enum meas_t { prep, B_shot }

    Measurement type.

Public Member Functions

- SingleSpin (const double &o=0.2, const double &deltao=0., const model_t &m=naiv, const meas_t &meas=prep, double B_meas=0., double tmin=0.)

  Constructor.
- virtual void Step ()

  Advances the simulation.
- void Print (std::ostream &out=std::cout)

  Prints the electron states at the scattering events.
- void RawPrint (std::ostream &out=std::cout)

  Prints the electron states at the scattering events without pretty formatting.
- arma::vec GetSpin (const double &t)

  Gets the electron spin state at an arbitrary time within the simulation range.
- void FillSzVec (std::vector< double > &Sz, const int &size, const double &dt)

  Fills a vector with a given spin component at uniform time samples.
- double GetFirstTime ()

  Gets the starting time of the simulation.
- double GetLastTime ()

  Gets the time of the last scattering event.

Protected Member Functions

- int **binary_search_t** (const double &t)

Protected Attributes

- model_t **model**
- meas_t **meas**
- double **tmin**
- arma::vec **B_meas**
- double **omega**
- double **delta_omega**
- std::vector< double > **times**
- std::vector< arma::vec > **kvecs**
- std::vector< arma::vec > **spins**

5.5.1 Detailed Description

Spin relaxation experiment class mainly for ensamble measurements.

It represents an experiment on a single spin. For an ensamble one has to create multiple classes an average over the results.

5.5.2 Member Enumeration Documentation

5.5.2.1 meas_t

```
enum SingleSpin::meas_t
```

Measurement type.

Enumerator

prep	Measurement starting from fully polarized spin state
B_shot	Measurement starting from random spin state, external magnetic field is turned on at t=0

5.5.2.2 model_t

`enum SingleSpin::model_t`

An enum type for the underlying Hamiltonian and direction of interest.

Each Hamiltonian has different built in SOC field, so they have different time evolutions. The direction of interest is relevant for the FillSzVec method. For a more detailed description of the models look at the main page.

Enumerator

naiv	Fully isotropic 3D model.
burkov_2d	2DEG model with Rashba SOC, z axis relaxation.
burkov_2d_Sx	2DEG model with Rashba SOC, x axis relaxationa.
burkov_2d_angle	2DEG model with Rashba SOC, spins started polarized at an angle to the z axis, z component gathered.
burkov_2d_angle_sx	2DEG model with Rashba SOC, spins started polarized at an angle to the z axis, x component gathered.
rashba_3d	3D model with Rashba SOC.
mixed_3d	3D model with both isotropic and Rashba SOC.
mn_1d	1D model which exactly gives back the result of motional narrowing.
dresselhaus	3D model with Dresselhaus SOC, z axis relaxation
dresselhaus_xy	3D model with Dresselhaus SOC, spins started with x direction polarization, y component gathered.
rashba_dressel_2d_z	2DEG model with both rashba and Dresselhaus SOC, z axis relaxation.
rashba_dressel_2d_x	2DEG model with both rashba and Dresselhaus SOC, x axis relaxation.
rashba_dressel_2d_xy	2DEG model with both rashba and Dresselhaus SOC, spins started with x direction polarization, y component gathered.
rashba_dressel_3d_x	3D model with both Dresselhaus and Rashba SOC, x axis relaxation.
rashba_dressel_3d_z	3D model with both Dresselhaus and Rashba SOC, z axis relaxation.
rashba_dressel_3d_xz	3D model with both Dresselhaus and Rashba SOC, spins started with x direction polarization, z component gathered.
rashba_dressel_3d_xy	3D model with both Dresselhaus and Rashba SOC, spins started with y direction polarization, y component gathered.
rashba_dressel_3d_111_xx	3D model with both Dresselhaus and Rashba SOC, [1,1,1] growth direction, x axis relaxation.
rashba_dressel_3d_111_zz	3D model with both Dresselhaus and Rashba SOC, [1,1,1] growth direction, z axis relaxation.

5.5.3 Constructor & Destructor Documentation

5.5.3.1 SingleSpin()

```
SingleSpin::SingleSpin (
            const double & o = 0.2,
            const double & deltao = 0.,
            const model_t & m = naiv,
            const meas_t & meas = prep,
            double B_meas = 0.,
            double tmin = 0. )
```

Constructor.

Parameters

o	The primary parameter for the SOC. It's typically the amplitude of the Larmor angular frequency due to the SOC field. The exact meaning depends on the model in question.
deltao	The secondary paramter for the SOC. It typically describes the deviation of amplitude of the Larmor angular frequency. The exact meaning is model dependent.
m	The model that selects the Hamiltonian, initial condition and the direction of interest.
meas	The measurement type.
B_meas	The amplitude of the external magnetic field for B_shot measurements.
tmin	The starting time of the measurement. The magnetic field is turned on at t=0 for B_shot measurements.

5.5.4 Member Function Documentation

5.5.4.1 FillSzVec()

```
void SingleSpin::FillSzVec (
            std::vector< double > & Sz,
            const int & size,
            const double & dt )
```

Fills a vector with a given spin component at uniform time samples.

Parameters

Sz	The vector to be filled.
size	The number of points gathered.
dt	The timestep between samples.

5.5.4.2 GetFirstTime()

```
double SingleSpin::GetFirstTime ( ) [inline]
```

Gets the starting time of the simulation.

Returns

The starting time of the simulation.

5.5.4.3 GetLastTime()

```
double SingleSpin::GetLastTime ( ) [inline]
```

Gets the time of the last scattering event.

Returns

The time of the last scattering event.

5.5.4.4 GetSpin()

```
arma::vec SingleSpin::GetSpin (
            const double & t )
```

Gets the electron spin state at an arbitrary time within the simulation range.

Returns

The electron spin state.

Exceptions

std::runtime_error	if t is not in range.

5.5.4.5 Step()

```
void SingleSpin::Step ( ) [virtual]
```

Advances the simulation.

The simulation is event driven. Each step advances the simulation to the next scattering event.

Reimplemented in SingleSpinAutocorr.

The documentation for this class was generated from the following files:

- include/singlespin.h
- src/singlespin.cpp

5.6 SingleSpinAutocorr Class Reference

Spin relaxation experiment class for autocorrelation measurements.

```
#include <singlespin.h>
```

Inheritance diagram for SingleSpinAutocorr:

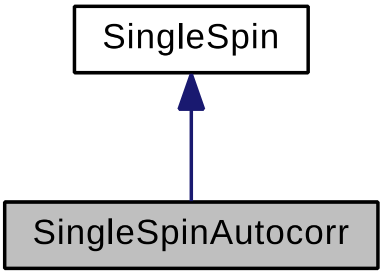

Collaboration diagram for SingleSpinAutocorr:

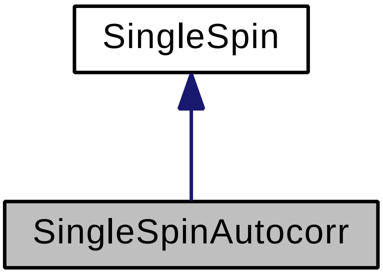

Public Member Functions

- SingleSpinAutocorr (const double &o, const double &deltao, const model_t &m, const meas_t &meas, double B_meas, double tmin, double dt, unsigned int N)

    Constructor.
- void Step ()

    Advances the simulation.
- std::unique_ptr< std::vector< double > > GetAutocorr ()

    Gets the autocorrelation vector gathered during the simulation.

Additional Inherited Members

5.6.1 Detailed Description

Spin relaxation experiment class for autocorrelation measurements.

It measures the spin-spin autocorrelation for a single spin. For a useful measurement it should be ran for a long time.

5.6.2 Constructor & Destructor Documentation

5.6.2.1 SingleSpinAutocorr()

```
SingleSpinAutocorr::SingleSpinAutocorr (
            const double & o,
            const double & deltao,
            const model_t & m,
            const meas_t & meas,
            double B_meas,
            double tmin,
            double dt,
            unsigned int N )
```

Constructor.

Parameters

o	The primary parameter for the SOC. It's typically the amplitude of the Larmor angular frequency due to the SOC field. The exact meaning depends on the model in question.
deltao	The secondary paramter for the SOC. It typically describes the deviation of amplitude of the Larmor angular frequency. The exact meaning is model dependent.
m	The model that selects the Hamiltonian, initial condition and the direction of interest.
meas	The measurement type.
B_meas	The amplitude of the external magnetic field for B_shot measurements.
tmin	The starting time of the measurement. The magnetic field is turned on at t=0 for B_shot measurements.
dt	The timestep for the autocorrelation data.
N	The number of samples for the autocorrelation data.

5.6.3 Member Function Documentation

5.6.3.1 GetAutocorr()

```
std::unique_ptr< std::vector< double > > SingleSpinAutocorr::GetAutocorr ( )
```

Gets the autocorrelation vector gathered during the simulation.

Returns

The autocorrelation vector.

5.6.3.2 Step()

```
void SingleSpinAutocorr::Step ( )  [virtual]
```

Advances the simulation.

The simulation is event driven. Each step advances the simulation to the next scattering event.

Reimplemented from SingleSpin.

The documentation for this class was generated from the following files:

- include/singlespin.h
- src/singlespin.cpp

Chapter 6

File Documentation

6.1 include/la.h File Reference

```
#include <armadillo>
```

Include dependency graph for la.h:

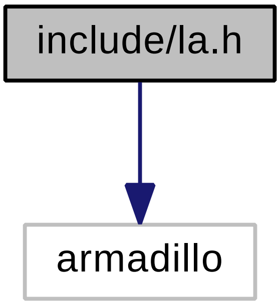

Functions

- arma::vec la::Rotate (const arma::vec &v0, const arma::vec &phi)

    Rotates a vector around an other vecor.

6.1.1 Function Documentation

6.1.1.1 Rotate()

```
arma::vec la::Rotate (
            const arma::vec & v0,
            const arma::vec & phi )
```

Rotates a vector around an other vecor.

Parameters

v0	The vector to be rotated.
phi	The angle vector. The length of this vector is the angle of the rotation in radians. The direction of the vector denotes the axis of the rotation. The rotation follows the right hand rule.

Returns

The rotated vector.

Index

autocorr, 11
    autocorr, 12
    get_autocorr, 12
    push, 12

buffer
    buffer, 13
    get_eff_size, 14
    operator[], 14
    push, 14
buffer< T >, 13

FillSzVec
    SingleSpin, 20

get_autocorr
    autocorr, 12
get_eff_size
    buffer, 14
GetAutocorr
    SingleSpinAutocorr, 23
GetFirstTime
    SingleSpin, 20
getGen
    randgen::gen, 15
GetLastTime
    SingleSpin, 21
GetSpin
    SingleSpin, 21

include/la.h, 25
Instance
    randgen::gen, 16

la.h
    Rotate, 25

meas_t
    SingleSpin, 18
model_t
    SingleSpin, 19

operator[]
    buffer, 14

Progress, 17
push
    autocorr, 12
    buffer, 14

randgen::gen, 15
    getGen, 15
    Instance, 16
Rotate
    la.h, 25

SingleSpin, 17
    FillSzVec, 20
    GetFirstTime, 20
    GetLastTime, 21
    GetSpin, 21
    meas_t, 18
    model_t, 19
    SingleSpin, 19
    Step, 21
SingleSpinAutocorr, 22
    GetAutocorr, 23
    SingleSpinAutocorr, 23
    Step, 23
Step
    SingleSpin, 21
    SingleSpinAutocorr, 23
