# Supplementary material for: Spin-relaxation time in materials with broken inversion symmetry and large spin-orbit coupling
Source: Sci Rep. 2017 Aug 30;7:9949. doi: 10.1038/s41598-017-09759-0 (PMC5577210; doi:10.1038/s41598-017-09759-0)
Supplement: Supplementary file 2 — The Monte Carlo code of the calculations in C++ [file 41598_2017_9759_MOESM2_ESM.zip › DP_Monte_Carlo/doc/html/functions_func.html]

Dyakonov Perel Monte Carlo simulation: Class Members - Functions


|  |
| --- |
| Dyakonov Perel Monte Carlo simulation |


- autocorr()
  : autocorr
- buffer()
  : buffer< T >
- FillSzVec()
  : SingleSpin
- get\_autocorr()
  : autocorr
- get\_eff\_size()
  : buffer< T >
- get\_size()
  : buffer< T >
- GetAutocorr()
  : SingleSpinAutocorr
- GetFirstTime()
  : SingleSpin
- getGen()
  : randgen::gen
- GetLastTime()
  : SingleSpin
- GetSpin()
  : SingleSpin
- Instance()
  : randgen::gen
- operator[]()
  : buffer< T >
- Print()
  : SingleSpin
- push()
  : autocorr
  , buffer< T >
- RawPrint()
  : SingleSpin
- SingleSpin()
  : SingleSpin
- SingleSpinAutocorr()
  : SingleSpinAutocorr
- Step()
  : SingleSpin
  , SingleSpinAutocorr


---

Generated by  

 1.8.13
