# Supplementary material for: Spin-relaxation time in materials with broken inversion symmetry and large spin-orbit coupling
Source: Sci Rep. 2017 Aug 30;7:9949. doi: 10.1038/s41598-017-09759-0 (PMC5577210; doi:10.1038/s41598-017-09759-0)
Supplement: Supplementary file 2 — The Monte Carlo code of the calculations in C++ [file 41598_2017_9759_MOESM2_ESM.zip › DP_Monte_Carlo/doc/html/random_8h_source.html]

Dyakonov Perel Monte Carlo simulation: include/random.h Source File


|  |
| --- |
| Dyakonov Perel Monte Carlo simulation |


- include

random.h

1 #include <boost/random.hpp>

2

3 namespace randgen{

4  typedef boost::random::mt19937 pseudogen;

5

13  class gen

14  {

15  public:

22  static gen\* Instance();

23

31  static gen\* Instance(uint32\_t seed);

32

39  pseudogen& getGen();

40  private:

41  pseudogen generator;

42  gen() {};

43  gen(uint32\_t seed): generator(seed) {};

44  gen(gen const& copy);

45  gen& operator=(gen const& copy);

46

47  static gen\* pInstance;

48  };

49 }

randgen

**Definition:** random.h:3

randgen::gen

Random generator singleton.

**Definition:** random.h:13

randgen::gen::getGen

pseudogen & getGen()

Gets the underlying random generator.

**Definition:** random.cpp:26

randgen::gen::Instance

static gen \* Instance()

Gets the unique instance of the random generator.

**Definition:** random.cpp:8


---

Generated by  

 1.8.13
