# Supplementary material for: Spin-relaxation time in materials with broken inversion symmetry and large spin-orbit coupling
Source: Sci Rep. 2017 Aug 30;7:9949. doi: 10.1038/s41598-017-09759-0 (PMC5577210; doi:10.1038/s41598-017-09759-0)
Supplement: Supplementary file 2 — The Monte Carlo code of the calculations in C++ [file 41598_2017_9759_MOESM2_ESM.zip › DP_Monte_Carlo/doc/html/classbuffer-members.html]

Dyakonov Perel Monte Carlo simulation: Member List


|  |
| --- |
| Dyakonov Perel Monte Carlo simulation |

buffer< T > Member List

This is the complete list of members for buffer< T >, including all inherited members.

|  |  |  |
| --- | --- | --- |
| buffer(size\_t size) | buffer< T > |  |
| get\_eff\_size() | buffer< T > | inline |
| get\_size() | buffer< T > | inline |
| operator[](size\_t idx) | buffer< T > |  |
| push(const T &value) | buffer< T > | virtual |


---

Generated by  

 1.8.13
