# Supplementary material for: Spin-relaxation time in materials with broken inversion symmetry and large spin-orbit coupling
Source: Sci Rep. 2017 Aug 30;7:9949. doi: 10.1038/s41598-017-09759-0 (PMC5577210; doi:10.1038/s41598-017-09759-0)
Supplement: Supplementary file 2 — The Monte Carlo code of the calculations in C++ [file 41598_2017_9759_MOESM2_ESM.zip › DP_Monte_Carlo/doc/html/singlespin_8h_source.html]

Dyakonov Perel Monte Carlo simulation: include/singlespin.h Source File


|  |
| --- |
| Dyakonov Perel Monte Carlo simulation |


- include

singlespin.h

1 #ifndef SINGLESPIN\_H

2 #define SINGLESPIN\_H

3

4 #include <armadillo>

5 #include <vector>

6 #include <iostream>

7 #include <memory>

8 #include "autocorr.h"

9

16 class SingleSpin {

17  public:

25  enum model\_t {

26  naiv,

27  burkov\_2d,

28  burkov\_2d\_Sx,

29  burkov\_2d\_angle,

30  burkov\_2d\_angle\_sx,

31  rashba\_3d,

32  mixed\_3d,

33  mn\_1d,

34  dresselhaus,

35  dresselhaus\_xy,

36  rashba\_dressel\_2d\_z,

37  rashba\_dressel\_2d\_x,

38  rashba\_dressel\_2d\_xy,

39  rashba\_dressel\_3d\_x,

40  rashba\_dressel\_3d\_z,

41  rashba\_dressel\_3d\_xz,

42  rashba\_dressel\_3d\_xy,

43  rashba\_dressel\_3d\_111\_xx,

44  rashba\_dressel\_3d\_111\_zz

45  };

49  enum meas\_t {

50  prep,

51  B\_shot

52  };

53  protected:

54  model\_t model;

55  meas\_t meas;

56  double tmin;

57

58  arma::vec B\_meas;

59  double omega;

60  double delta\_omega;

61  std::vector<double> times;

62  std::vector<arma::vec> kvecs;

63  std::vector<arma::vec> spins;

64  int binary\_search\_t(const double &t);

65  public:

66

82  SingleSpin(const double& o=0.2,

83  const double& deltao=0.,

84  const model\_t& m=naiv,

85  const meas\_t& meas=prep,

86  double B\_meas=0.,

87  double tmin=0.);

88

95  virtual void Step();

96

101  void Print(std::ostream& out=std::cout);

102

105  void RawPrint(std::ostream& out=std::cout);

106

112  arma::vec GetSpin(const double &t);

113

121  void FillSzVec(std::vector<double>& Sz, const int& size, const double& dt);

122

127  double GetFirstTime() {return times.front();}

128

133  double GetLastTime() {return times.back(); }

134 };

135

142 class SingleSpinAutocorr : public SingleSpin {

143  private:

144  autocorr buf;

145  const double dt;

146  public:

153  SingleSpinAutocorr(const double& o,

154  const double& deltao,

155  const model\_t& m,

156  const meas\_t& meas,

157  double B\_meas,

158  double tmin,

159  double dt,

160  unsigned int N

161  );

162  void Step();

163

169  std::unique\_ptr<std::vector<double>> GetAutocorr();

170 };

171

172 #endif

SingleSpin::rashba\_3d

**Definition:** singlespin.h:31

SingleSpin::naiv

**Definition:** singlespin.h:26

SingleSpin

Spin relaxation experiment class mainly for ensamble measurements.

**Definition:** singlespin.h:16

SingleSpin::rashba\_dressel\_3d\_xz

**Definition:** singlespin.h:41

SingleSpin::RawPrint

void RawPrint(std::ostream &out=std::cout)

Prints the electron states at the scattering events without pretty formatting.

**Definition:** singlespin.cpp:363

SingleSpin::burkov\_2d\_angle

**Definition:** singlespin.h:29

SingleSpin::rashba\_dressel\_3d\_111\_xx

**Definition:** singlespin.h:43

SingleSpin::FillSzVec

void FillSzVec(std::vector< double > &Sz, const int &size, const double &dt)

Fills a vector with a given spin component at uniform time samples.

**Definition:** singlespin.cpp:421

SingleSpin::burkov\_2d\_Sx

**Definition:** singlespin.h:28

SingleSpin::rashba\_dressel\_3d\_111\_zz

**Definition:** singlespin.h:44

SingleSpinAutocorr

Spin relaxation experiment class for autocorrelation measurements.

**Definition:** singlespin.h:142

autocorr

Class for gathering autocorrelation of time series data.

**Definition:** autocorr.h:13

SingleSpin::rashba\_dressel\_2d\_z

**Definition:** singlespin.h:36

SingleSpin::dresselhaus\_xy

**Definition:** singlespin.h:35

SingleSpin::Step

virtual void Step()

Advances the simulation.

**Definition:** singlespin.cpp:206

SingleSpin::rashba\_dressel\_3d\_z

**Definition:** singlespin.h:40

SingleSpin::rashba\_dressel\_3d\_x

**Definition:** singlespin.h:39

SingleSpin::burkov\_2d

**Definition:** singlespin.h:27

SingleSpin::mn\_1d

**Definition:** singlespin.h:33

SingleSpin::model\_t

model\_t

An enum type for the underlying Hamiltonian and direction of interest.

**Definition:** singlespin.h:25

SingleSpin::rashba\_dressel\_2d\_x

**Definition:** singlespin.h:37

SingleSpin::GetLastTime

double GetLastTime()

Gets the time of the last scattering event.

**Definition:** singlespin.h:133

SingleSpin::B\_shot

**Definition:** singlespin.h:51

SingleSpin::rashba\_dressel\_2d\_xy

**Definition:** singlespin.h:38

SingleSpin::Print

void Print(std::ostream &out=std::cout)

Prints the electron states at the scattering events.

**Definition:** singlespin.cpp:352

SingleSpin::rashba\_dressel\_3d\_xy

**Definition:** singlespin.h:42

SingleSpin::prep

**Definition:** singlespin.h:50

SingleSpin::meas\_t

meas\_t

Measurement type.

**Definition:** singlespin.h:49

SingleSpin::mixed\_3d

**Definition:** singlespin.h:32

SingleSpin::burkov\_2d\_angle\_sx

**Definition:** singlespin.h:30

SingleSpin::dresselhaus

**Definition:** singlespin.h:34

SingleSpin::GetSpin

arma::vec GetSpin(const double &t)

Gets the electron spin state at an arbitrary time within the simulation range.

**Definition:** singlespin.cpp:376

SingleSpin::SingleSpin

SingleSpin(const double &o=0.2, const double &deltao=0., const model\_t &m=naiv, const meas\_t &meas=prep, double B\_meas=0., double tmin=0.)

Constructor.

**Definition:** singlespin.cpp:10

SingleSpin::GetFirstTime

double GetFirstTime()

Gets the starting time of the simulation.

**Definition:** singlespin.h:127


---

Generated by  

 1.8.13
