# Supplementary material for: Spin-relaxation time in materials with broken inversion symmetry and large spin-orbit coupling
Source: Sci Rep. 2017 Aug 30;7:9949. doi: 10.1038/s41598-017-09759-0 (PMC5577210; doi:10.1038/s41598-017-09759-0)
Supplement: Supplementary file 2 — The Monte Carlo code of the calculations in C++ [file 41598_2017_9759_MOESM2_ESM.zip › DP_Monte_Carlo/doc/html/classbuffer.html]

Dyakonov Perel Monte Carlo simulation: buffer< T > Class Template Reference


|  |
| --- |
| Dyakonov Perel Monte Carlo simulation |

Public Member Functions |
List of all members

buffer< T > Class Template Reference

A circular buffer template class.
More...

`#include <buffer.h>`

|  |  |
| --- | --- |
| Public Member Functions | |
|  | buffer (size\_t size) |
|  | Constructor. More... |
|  | |
| T & | operator[] (size\_t idx) |
|  | Access specified element. More... |
|  | |
| virtual void | push (const T &value) |
|  | Pushes an element. More... |
|  | |
| size\_t | get\_size () |
|  | Gets the allocated size of the buffer. |
|  | |
| size\_t | get\_eff\_size () |
|  | Gets the apparent size of the buffer. More... |
|  | |

## Detailed Description

### template<typename T> class buffer< T >

A circular buffer template class.

This class acts like std::vector, except it "forgets" pushed elements that no longor fit inside.

## Constructor & Destructor Documentation

## ◆ buffer()

template<typename T >

|  |  |  |  |  |  |
| --- | --- | --- | --- | --- | --- |
| buffer< T >::buffer | ( | size\_t | *size* | ) |  |

Constructor.

Parameters
:   |  |  |
    | --- | --- |
    | size | The allocated size of the buffer. |

## Member Function Documentation

## ◆ get\_eff\_size()

template<typename T>

|  |  |  |  |  |  |  |
| --- | --- | --- | --- | --- | --- | --- |
| |  |  |  |  |  | | --- | --- | --- | --- | --- | | size\_t buffer< T >::get\_eff\_size | ( |  | ) |  | | inline |

Gets the apparent size of the buffer.

The apparent size increases every time an element is pushed.

## ◆ operator[]()

template<typename T >

|  |  |  |  |  |  |
| --- | --- | --- | --- | --- | --- |
| T & buffer< T >::operator[] | ( | size\_t | *idx* | ) |  |

Access specified element.

Parameters
:   |  |  |
    | --- | --- |
    | idx | Index of the given element. |

Returns
:   The specified element.

Exceptions
:   |  |  |
    | --- | --- |
    | std::out\_of\_range |  |

The maximal idx available increases for every push() operation. After the buffer gets full, then the minimal available index increases as well. If idx is not in legal range it throws std::out\_of\_range.

## ◆ push()

template<typename T>

|  |  |  |  |  |  |  |  |
| --- | --- | --- | --- | --- | --- | --- | --- |
| |  |  |  |  |  |  | | --- | --- | --- | --- | --- | --- | | void buffer< T >::push | ( | const T & | *value* | ) |  | | virtual |

Pushes an element.

Parameters
:   |  |  |
    | --- | --- |
    | value | The pushed element. |

The pushed element will be available at object[get\_eff\_size()-1].

Reimplemented in autocorr.

---

The documentation for this class was generated from the following file:

- include/buffer.h


---

Generated by  

 1.8.13
