# Supplementary material for: Spin-relaxation time in materials with broken inversion symmetry and large spin-orbit coupling
Source: Sci Rep. 2017 Aug 30;7:9949. doi: 10.1038/s41598-017-09759-0 (PMC5577210; doi:10.1038/s41598-017-09759-0)
Supplement: Supplementary file 2 — The Monte Carlo code of the calculations in C++ [file 41598_2017_9759_MOESM2_ESM.zip › DP_Monte_Carlo/doc/html/index.html]

Dyakonov Perel Monte Carlo simulation: Main Page


|  |
| --- |
| Dyakonov Perel Monte Carlo simulation |


Dyakonov Perel Monte Carlo simulation Documentation

# Compilation

The program is mainly developed on a Linux Debian distribution. Compilation depends on the *libarmadillo-dev* *libboost-program-options-dev* and *libboost-random-dev* packages. Running a single *make* command compiles the program to the path *bin/main*.

# Usage

The program can be operated with command line options, it allows scripting large number of simulations with different parameters.

$ bin/main --help

Allowed options:

-h [ --help ] produce help message

-v [ --version ] print version number

--autocorr set autocorr measurement

--spins arg (=10) set number of spins

--duration arg (=300) set simulation duration

--timestep arg (=1) set timestep

--omega arg (=0.20000000000000001) set the absolute value of Larmor

precession

--delta\_omega arg (=0) set the width of omega distribution

--seed arg (=rand) set the seed for the random generator

-o [ --output ] arg (=-) output file path

-m [ --model ] arg (=naiv) name of the model

--meas arg (=prep) name of measurement method

-b [ --B\_meas ] arg (=0) measurement field

--tmin arg (=0) starting time, B\_meas turns in at t=0

As there are default arguments for all options (as seen in the help output), with no arguments the program outputs the result of a sample simulation. The output contains a header which contains the simulation parameters so the simulation can be reproduced. After the header it outputs the spin component of interest in the function of time. The time interval and sampling interval is the same as specified in the command line.

$ bin/main | head -20

# Djakonov-Perel simulation

# t=0 Sz=1, no magnetic field

# version: commit\_17cc1d6e5275608347fa311f7f7e64735470c311

# spins: 10

# duration: 300

# timestep: 1

# omega: 0.2

# seed: 2494990189

# model: naiv

# meas: prep

# B\_meas: 0

# tmin: 0

# autocorr: false

# t, Sz

0, 1

1, 0.990826

2, 0.97055

3, 0.946542

4, 0.925218

5, 0.910056

# Command line options

We can see from the output of "bin/main --help" that there are many options regarding to a single run of the simulation. Here I describe the details how these options work.

- **help** Outputs the brief description of command line options.
- **version** Outputs the version number. If the program is compiled in a clean state git repository then it is the commit hash of the given version.
- **duration** Sets the simulation time window's length.
- **timestep** Sets the interval between time samples.
- **tmin** Sets the starting time of the measurement, it is useful for measurements with step function external magnetic field, where it is turned on at t=0.
- **omega** The primary parameter of the underlying SOC model. It is typically the amplitude of the Larmor angular frequency due to the SOC field. The exact meaning depends on the model in question.
- **delta\_omega** The secondary paramter for the SOC. It typically describes the deviation of amplitude of the Larmor angular frequency. The exact meaning is model dependent.
- **model** The name of the spin model selected. It also selects the initial condition and the spin direction of interest. The Hamiltonian has the form:

  In all models the exact  are paremetrized by two parameters,  and . These can be set by the options **omega** and **delta\_omega** respectively.

  The available models:

  - *naiv* Fully isotropic 3D SOC model, we assume a spherical Fermi-surface.
  - *burkov\_2d* 2DEG model with Rashba SOC, z axis relaxation. The shape of the Fermi surface is a circle.
  - *burkov\_2d\_Sx* 2DEG model with Rashba SOC, x axis relaxation.
  - *burkov\_2d\_angle* 2DEG model with Rashba SOC, spins started polarized at a 45° angle to the z axis, z component gathered.
  - *burkov\_2d\_angle\_sx* 2DEG model with Rashba SOC, spins started polarized at a 45° angle to the z axis, x component gathered.
  - *rashba\_3d* 3D model with Rashba SOC. The SOC Hamiltonian is the same as in *burkov\_2d*, but now we allow wave numbers in the z direction. We assume a spherical Fermi-surface.
  - *mixed\_3d* 3D model with both isotropic and Rashba SOC.
  - *mn\_1d* 1D model which exactly gives back the result of motional narrowing. The "Fermi-surface" is two points , . We start the spin from the z axis, the SOC field is perpendicular to the z axis.
  - *dresselhaus* 3D model with dresselhaus SOC, z axis relaxation.
  - *dresselhaus\_xy* 3D model with dresselhaus SOC, spins started with x direction polarization, y component gathered.
  - *rashba\_dressel\_2d\_z* 2DEG model with both rashba and dresselhaus SOC, z axis relaxation.
  - *rashba\_dressel\_2d\_x* 2DEG model with both rashba and dresselhaus SOC, x axis relaxation.
  - *rashba\_dressel\_2d\_xy* 2DEG model with both rashba and dresselhaus SOC, spins started with x direction polarization, y component gathered.
  - *rashba\_dressel\_3d\_x* 3D model with both Dresselhaus and Rashba SOC, x axis relaxation. We assume [0,0,1] growth direction.
  - *rashba\_dressel\_3d\_z* 3D model with both Dresselhaus and Rashba SOC, z axis relaxation.
  - *rashba\_dressel\_3d\_xz* 3D model with both Dresselhaus and Rashba SOC, spins started with x direction polarization, z component gathered.
  - *rashba\_dressel\_3d\_xy* 3D model with both Dresselhaus and Rashba SOC, spins started with y direction polarization, y component gathered.
  - *rashba\_dressel\_3d\_111\_xx* 3D model with both Dresselhaus and Rashba SOC, [1,1,1] growth direction, x axis relaxation. The coordinate system's z axis is aligned to the growth direction.
  - *rashba\_dressel\_3d\_111\_zz* 3D model with both Dresselhaus and Rashba SOC, [1,1,1] growth direction, z axis relaxation.
- **autocorr**: If this option is set, then <s(t+tau)s(t)> time averaged autocorrelation is measured for a single spin. At this point the meaning of the options **spins** and **duration** changes. **duration** becomes the time window from where we take the tau values, **timestep** is the sampling interval for the **tau** values. The simulation is actually ran for **duration** × **spins** time, so the resulting graph has similar errors as an ensamble measurement.
- **seed** The random seed of the random generator. Potentially useful for debugging as a fix value guarantees deterministic runs.
- **output** The output file for the simulation, default is stdout.


---

Generated by  

 1.8.13
