# Supplementary material for: Spin-relaxation time in materials with broken inversion symmetry and large spin-orbit coupling
Source: Sci Rep. 2017 Aug 30;7:9949. doi: 10.1038/s41598-017-09759-0 (PMC5577210; doi:10.1038/s41598-017-09759-0)
Supplement: Supplementary file 2 — The Monte Carlo code of the calculations in C++ [file 41598_2017_9759_MOESM2_ESM.zip › DP_Monte_Carlo/doc/html/functions.html]

Dyakonov Perel Monte Carlo simulation: Class Members


|  |
| --- |
| Dyakonov Perel Monte Carlo simulation |


Here is a list of all documented class members with links to the class documentation for each member:

### - a -

- autocorr()
  : autocorr

### - b -

- B\_shot
  : SingleSpin
- buffer()
  : buffer< T >
- burkov\_2d
  : SingleSpin
- burkov\_2d\_angle
  : SingleSpin
- burkov\_2d\_angle\_sx
  : SingleSpin
- burkov\_2d\_Sx
  : SingleSpin

### - d -

- dresselhaus
  : SingleSpin
- dresselhaus\_xy
  : SingleSpin

### - f -

- FillSzVec()
  : SingleSpin

### - g -

- get\_autocorr()
  : autocorr
- get\_eff\_size()
  : buffer< T >
- get\_size()
  : buffer< T >
- GetAutocorr()
  : SingleSpinAutocorr
- GetFirstTime()
  : SingleSpin
- getGen()
  : randgen::gen
- GetLastTime()
  : SingleSpin
- GetSpin()
  : SingleSpin

### - i -

- Instance()
  : randgen::gen

### - m -

- meas\_t
  : SingleSpin
- mixed\_3d
  : SingleSpin
- mn\_1d
  : SingleSpin
- model\_t
  : SingleSpin

### - n -

- naiv
  : SingleSpin

### - o -

- operator[]()
  : buffer< T >

### - p -

- prep
  : SingleSpin
- Print()
  : SingleSpin
- push()
  : autocorr
  , buffer< T >

### - r -

- rashba\_3d
  : SingleSpin
- rashba\_dressel\_2d\_x
  : SingleSpin
- rashba\_dressel\_2d\_xy
  : SingleSpin
- rashba\_dressel\_2d\_z
  : SingleSpin
- rashba\_dressel\_3d\_111\_xx
  : SingleSpin
- rashba\_dressel\_3d\_111\_zz
  : SingleSpin
- rashba\_dressel\_3d\_x
  : SingleSpin
- rashba\_dressel\_3d\_xy
  : SingleSpin
- rashba\_dressel\_3d\_xz
  : SingleSpin
- rashba\_dressel\_3d\_z
  : SingleSpin
- RawPrint()
  : SingleSpin

### - s -

- SingleSpin()
  : SingleSpin
- SingleSpinAutocorr()
  : SingleSpinAutocorr
- Step()
  : SingleSpin
  , SingleSpinAutocorr


---

Generated by  

 1.8.13
