# Supplementary material for: Spin-relaxation time in materials with broken inversion symmetry and large spin-orbit coupling
Source: Sci Rep. 2017 Aug 30;7:9949. doi: 10.1038/s41598-017-09759-0 (PMC5577210; doi:10.1038/s41598-017-09759-0)
Supplement: Supplementary file 2 — The Monte Carlo code of the calculations in C++ [file 41598_2017_9759_MOESM2_ESM.zip › DP_Monte_Carlo/doc/html/hierarchy.html]

Dyakonov Perel Monte Carlo simulation: Class Hierarchy


|  |
| --- |
| Dyakonov Perel Monte Carlo simulation |


Class Hierarchy

Go to the graphical class hierarchy

This inheritance list is sorted roughly, but not completely, alphabetically:

[detail level 12]

|  |  |
| --- | --- |
| Cbuffer< T > | A circular buffer template class |
| ▼Cbuffer< double > |  |
| Cautocorr | Class for gathering autocorrelation of time series data |
| Crandgen::gen | Random generator singleton |
| CProgress |  |
| ▼CSingleSpin | Spin relaxation experiment class mainly for ensamble measurements |
| CSingleSpinAutocorr | Spin relaxation experiment class for autocorrelation measurements |


---

Generated by  

 1.8.13
