# Supplementary material for: Spin-relaxation time in materials with broken inversion symmetry and large spin-orbit coupling
Source: Sci Rep. 2017 Aug 30;7:9949. doi: 10.1038/s41598-017-09759-0 (PMC5577210; doi:10.1038/s41598-017-09759-0)
Supplement: Supplementary file 2 — The Monte Carlo code of the calculations in C++ [file 41598_2017_9759_MOESM2_ESM.zip › DP_Monte_Carlo/doc/html/classSingleSpinAutocorr.html]

Dyakonov Perel Monte Carlo simulation: SingleSpinAutocorr Class Reference


|  |
| --- |
| Dyakonov Perel Monte Carlo simulation |

Public Member Functions |
List of all members

SingleSpinAutocorr Class Reference

Spin relaxation experiment class for autocorrelation measurements.
More...

`#include <singlespin.h>`

Inheritance diagram for SingleSpinAutocorr:

<p><b>This browser is not able to show SVG: try Firefox, Chrome, Safari, or Opera instead.</b></p>

[legend]

Collaboration diagram for SingleSpinAutocorr:

<p><b>This browser is not able to show SVG: try Firefox, Chrome, Safari, or Opera instead.</b></p>

[legend]

|  |  |
| --- | --- |
| Public Member Functions | |
|  | SingleSpinAutocorr (const double &o, const double &deltao, const model\_t &m, const meas\_t &meas, double B\_meas, double tmin, double dt, unsigned int N) |
|  | Constructor. More... |
|  | |
| void | Step () |
|  | Advances the simulation. More... |
|  | |
| std::unique\_ptr< std::vector< double > > | GetAutocorr () |
|  | Gets the autocorrelation vector gathered during the simulation. More... |
|  | |
| Public Member Functions inherited from SingleSpin | |
|  | SingleSpin (const double &o=0.2, const double &deltao=0., const model\_t &m=naiv, const meas\_t &meas=prep, double B\_meas=0., double tmin=0.) |
|  | Constructor. More... |
|  | |
| void | Print (std::ostream &out=std::cout) |
|  | Prints the electron states at the scattering events. |
|  | |
| void | RawPrint (std::ostream &out=std::cout) |
|  | Prints the electron states at the scattering events without pretty formatting. |
|  | |
| arma::vec | GetSpin (const double &t) |
|  | Gets the electron spin state at an arbitrary time within the simulation range. More... |
|  | |
| void | FillSzVec (std::vector< double > &Sz, const int &size, const double &dt) |
|  | Fills a vector with a given spin component at uniform time samples. More... |
|  | |
| double | GetFirstTime () |
|  | Gets the starting time of the simulation. More... |
|  | |
| double | GetLastTime () |
|  | Gets the time of the last scattering event. More... |
|  | |

|  |  |
| --- | --- |
| Additional Inherited Members | |
| Public Types inherited from SingleSpin | |
| enum | model\_t {     naiv, burkov\_2d, burkov\_2d\_Sx, burkov\_2d\_angle,     burkov\_2d\_angle\_sx, rashba\_3d, mixed\_3d, mn\_1d,     dresselhaus, dresselhaus\_xy, rashba\_dressel\_2d\_z, rashba\_dressel\_2d\_x,     rashba\_dressel\_2d\_xy, rashba\_dressel\_3d\_x, rashba\_dressel\_3d\_z, rashba\_dressel\_3d\_xz,     rashba\_dressel\_3d\_xy, rashba\_dressel\_3d\_111\_xx, rashba\_dressel\_3d\_111\_zz   }|  | An enum type for the underlying Hamiltonian and direction of interest. More... | |
|  | |
| enum | meas\_t { prep, B\_shot }|  | Measurement type. More... | |
|  | |
| Protected Member Functions inherited from SingleSpin | |
| int | **binary\_search\_t** (const double &t) |
|  | |
| Protected Attributes inherited from SingleSpin | |
| model\_t | **model** |
|  | |
| meas\_t | **meas** |
|  | |
| double | **tmin** |
|  | |
| arma::vec | **B\_meas** |
|  | |
| double | **omega** |
|  | |
| double | **delta\_omega** |
|  | |
| std::vector< double > | **times** |
|  | |
| std::vector< arma::vec > | **kvecs** |
|  | |
| std::vector< arma::vec > | **spins** |
|  | |

## Detailed Description

Spin relaxation experiment class for autocorrelation measurements.

It measures the spin-spin autocorrelation for a single spin. For a useful measurement it should be ran for a long time.

## Constructor & Destructor Documentation

## ◆ SingleSpinAutocorr()

|  |  |  |  |
| --- | --- | --- | --- |
| SingleSpinAutocorr::SingleSpinAutocorr | ( | const double & | *o*, |
|  |  | const double & | *deltao*, |
|  |  | const model\_t & | *m*, |
|  |  | const meas\_t & | *meas*, |
|  |  | double | *B\_meas*, |
|  |  | double | *tmin*, |
|  |  | double | *dt*, |
|  |  | unsigned int | *N* |
|  | ) |  |  |

Constructor.

Parameters
:   |  |  |
    | --- | --- |
    | o | The primary parameter for the SOC. It's typically the amplitude of the Larmor angular frequency due to the SOC field. The exact meaning depends on the model in question. |
    | deltao | The secondary paramter for the SOC. It typically describes the deviation of amplitude of the Larmor angular frequency. The exact meaning is model dependent. |
    | m | The model that selects the Hamiltonian, initial condition and the direction of interest. |
    | meas | The measurement type. |
    | B\_meas | The amplitude of the external magnetic field for B\_shot measurements. |
    | tmin | The starting time of the measurement. The magnetic field is turned on at t=0 for B\_shot measurements. |
    | dt | The timestep for the autocorrelation data. |
    | N | The number of samples for the autocorrelation data. |

## Member Function Documentation

## ◆ GetAutocorr()

|  |  |  |  |  |
| --- | --- | --- | --- | --- |
| std::unique\_ptr< std::vector< double > > SingleSpinAutocorr::GetAutocorr | ( |  | ) |  |

Gets the autocorrelation vector gathered during the simulation.

Returns
:   The autocorrelation vector.

## ◆ Step()

|  |  |  |  |  |  |  |
| --- | --- | --- | --- | --- | --- | --- |
| |  |  |  |  |  | | --- | --- | --- | --- | --- | | void SingleSpinAutocorr::Step | ( |  | ) |  | | virtual |

Advances the simulation.

The simulation is event driven. Each step advances the simulation to the next scattering event.

Reimplemented from SingleSpin.

---

The documentation for this class was generated from the following files:

- include/singlespin.h
- src/singlespin.cpp


---

Generated by  

 1.8.13
