# Supplementary material for: Spin-relaxation time in materials with broken inversion symmetry and large spin-orbit coupling
Source: Sci Rep. 2017 Aug 30;7:9949. doi: 10.1038/s41598-017-09759-0 (PMC5577210; doi:10.1038/s41598-017-09759-0)
Supplement: Supplementary file 2 — The Monte Carlo code of the calculations in C++ [file 41598_2017_9759_MOESM2_ESM.zip › DP_Monte_Carlo/doc/html/classrandgen_1_1gen.html]

Dyakonov Perel Monte Carlo simulation: randgen::gen Class Reference


|  |
| --- |
| Dyakonov Perel Monte Carlo simulation |


- **randgen**
- gen

Public Member Functions |
Static Public Member Functions |
List of all members

randgen::gen Class Reference

Random generator singleton.
More...

`#include <random.h>`

|  |  |
| --- | --- |
| Public Member Functions | |
| pseudogen & | getGen () |
|  | Gets the underlying random generator. More... |
|  | |

|  |  |
| --- | --- |
| Static Public Member Functions | |
| static gen \* | Instance () |
|  | Gets the unique instance of the random generator. More... |
|  | |
| static gen \* | Instance (uint32\_t seed) |
|  | Gets the unique instance of the random generator. More... |
|  | |

## Detailed Description

Random generator singleton.

The random generator singleton used throughout the codebase. It prevents the creation of multiple independent generators, which could potentially hurt the randomness of the simulation.

## Member Function Documentation

## ◆ getGen()

|  |  |  |  |  |
| --- | --- | --- | --- | --- |
| pseudogen & randgen::gen::getGen | ( |  | ) |  |

Gets the underlying random generator.

Returns
:   The underlying random generator.

The returntype is pseudogen typedefd to boost::random::mt19937.

## ◆ Instance() [1/2]

|  |  |  |  |  |  |  |
| --- | --- | --- | --- | --- | --- | --- |
| |  |  |  |  |  | | --- | --- | --- | --- | --- | | gen \* randgen::gen::Instance | ( |  | ) |  | | static |

Gets the unique instance of the random generator.

Returns
:   Pointer to the instance.

It constructs the generator with a random seed (using the OS's entropy pool) if it wasn't already constructed.

## ◆ Instance() [2/2]

|  |  |  |  |  |  |  |  |
| --- | --- | --- | --- | --- | --- | --- | --- |
| |  |  |  |  |  |  | | --- | --- | --- | --- | --- | --- | | gen \* randgen::gen::Instance | ( | uint32\_t | *seed* | ) |  | | static |

Gets the unique instance of the random generator.

Parameters
:   |  |  |
    | --- | --- |
    | seed | The seed passed to the constructor of the random generator. |

Returns
:   Pointer to the instance.

The seed parameter is omitted if the instance already exists.

---

The documentation for this class was generated from the following files:

- include/random.h
- src/random.cpp


---

Generated by  

 1.8.13
