# Supplementary material for: Spin-relaxation time in materials with broken inversion symmetry and large spin-orbit coupling
Source: Sci Rep. 2017 Aug 30;7:9949. doi: 10.1038/s41598-017-09759-0 (PMC5577210; doi:10.1038/s41598-017-09759-0)
Supplement: Supplementary file 2 — The Monte Carlo code of the calculations in C++ [file 41598_2017_9759_MOESM2_ESM.zip › DP_Monte_Carlo/doc/html/buffer_8h_source.html]

Dyakonov Perel Monte Carlo simulation: include/buffer.h Source File


|  |
| --- |
| Dyakonov Perel Monte Carlo simulation |


- include

buffer.h

1 #ifndef BUFFER\_H

2 #define BUFFER\_H

3 #include<vector>

4 #include<stdexcept>

5

12 template<typename T>

13 class buffer{

14  private:

15  std::vector<T> container;

16  size\_t eff\_size;

17  size\_t size;

18

19  public:

20

26  buffer(size\_t size);

27

40  T& operator[](size\_t idx);

41

49  virtual void push(const T& value);

50

54  inline size\_t get\_size() { return size; }

55

61  inline size\_t get\_eff\_size() { return eff\_size; }

62 };

63

64 template<typename T>

65 buffer<T>::buffer(size\_t size): container(size), size(size), eff\_size(0)

66 {

67 }

68

69 template<typename T>

70 T& buffer<T>::operator[](size\_t idx)

71 {

72  if ( idx >= eff\_size )

73  throw std::out\_of\_range("Buffer over-index");

74  if ( eff\_size >= size )

75  if ( idx < eff\_size-size )

76  throw std::out\_of\_range("Buffer under-index");

77

78  return container[idx % size];

79 }

80

81 template<typename T>

82 void buffer<T>::push(const T& item)

83 {

84  eff\_size++;

85  this->operator[](eff\_size-1)=item;

86 }

87

88 #endif

buffer::get\_size

size\_t get\_size()

Gets the allocated size of the buffer.

**Definition:** buffer.h:54

buffer::get\_eff\_size

size\_t get\_eff\_size()

Gets the apparent size of the buffer.

**Definition:** buffer.h:61

buffer

A circular buffer template class.

**Definition:** buffer.h:13

buffer::buffer

buffer(size\_t size)

Constructor.

**Definition:** buffer.h:65

buffer::push

virtual void push(const T &value)

Pushes an element.

**Definition:** buffer.h:82

buffer::operator[]

T & operator[](size\_t idx)

Access specified element.

**Definition:** buffer.h:70


---

Generated by  

 1.8.13
