# Supplementary material for: Spin-relaxation time in materials with broken inversion symmetry and large spin-orbit coupling
Source: Sci Rep. 2017 Aug 30;7:9949. doi: 10.1038/s41598-017-09759-0 (PMC5577210; doi:10.1038/s41598-017-09759-0)
Supplement: Supplementary file 2 — The Monte Carlo code of the calculations in C++ [file 41598_2017_9759_MOESM2_ESM.zip › DP_Monte_Carlo/doc/html/autocorr_8h_source.html]

Dyakonov Perel Monte Carlo simulation: include/autocorr.h Source File


|  |
| --- |
| Dyakonov Perel Monte Carlo simulation |


- include

autocorr.h

1 #ifndef AUTOCORR\_H

2 #define AUTOCORR\_H

3

4 #include<vector>

5 #include<memory>

6 #include "buffer.h"

7

13 class autocorr : public buffer<double>{

14  private:

15  std::vector<double> autocorrsum;

16  std::vector<int> autocorrcount;

17  public:

21  autocorr(size\_t size);

22  void push(const double& value);

31  std::unique\_ptr<std::vector<double> > get\_autocorr();

32 };

33 #endif

autocorr

Class for gathering autocorrelation of time series data.

**Definition:** autocorr.h:13

buffer

A circular buffer template class.

**Definition:** buffer.h:13

autocorr::get\_autocorr

std::unique\_ptr< std::vector< double > > get\_autocorr()

Gets the autocorrelation vector.

**Definition:** autocorr.cpp:24

autocorr::push

void push(const double &value)

Pushes an element.

**Definition:** autocorr.cpp:14

autocorr::autocorr

autocorr(size\_t size)

Constructor.

**Definition:** autocorr.cpp:8


---

Generated by  

 1.8.13
