# Supplementary material for: Spin-relaxation time in materials with broken inversion symmetry and large spin-orbit coupling
Source: Sci Rep. 2017 Aug 30;7:9949. doi: 10.1038/s41598-017-09759-0 (PMC5577210; doi:10.1038/s41598-017-09759-0)
Supplement: Supplementary file 2 — The Monte Carlo code of the calculations in C++ [file 41598_2017_9759_MOESM2_ESM.zip › DP_Monte_Carlo/doc/html/classautocorr.html]

Dyakonov Perel Monte Carlo simulation: autocorr Class Reference


|  |
| --- |
| Dyakonov Perel Monte Carlo simulation |

Public Member Functions |
List of all members

autocorr Class Reference

Class for gathering autocorrelation of time series data.
More...

`#include <autocorr.h>`

Inheritance diagram for autocorr:

<p><b>This browser is not able to show SVG: try Firefox, Chrome, Safari, or Opera instead.</b></p>

[legend]

Collaboration diagram for autocorr:

<p><b>This browser is not able to show SVG: try Firefox, Chrome, Safari, or Opera instead.</b></p>

[legend]

|  |  |
| --- | --- |
| Public Member Functions | |
|  | autocorr (size\_t size) |
|  | Constructor. More... |
|  | |
| void | push (const double &value) |
|  | Pushes an element. More... |
|  | |
| std::unique\_ptr< std::vector< double > > | get\_autocorr () |
|  | Gets the autocorrelation vector. More... |
|  | |
| Public Member Functions inherited from buffer< double > | |
|  | buffer (size\_t size) |
|  | Constructor. More... |
|  | |
| double & | operator[] (size\_t idx) |
|  | Access specified element. More... |
|  | |
| size\_t | get\_size () |
|  | Gets the allocated size of the buffer. |
|  | |
| size\_t | get\_eff\_size () |
|  | Gets the apparent size of the buffer. More... |
|  | |

## Detailed Description

Class for gathering autocorrelation of time series data.

For a time series a\_0, a\_1, a\_2 ... it collects the mean values <a\_i\*a\_i>, <a\_(i+1)\*a\_i>, <a\_(i+2)\*a\_i> ...

## Constructor & Destructor Documentation

## ◆ autocorr()

|  |  |  |  |  |  |
| --- | --- | --- | --- | --- | --- |
| autocorr::autocorr | ( | size\_t | *size* | ) |  |

Constructor.

Parameters
:   |  |  |
    | --- | --- |
    | size | The allocated size of the buffer. |

## Member Function Documentation

## ◆ get\_autocorr()

|  |  |  |  |  |
| --- | --- | --- | --- | --- |
| std::unique\_ptr< std::vector< double > > autocorr::get\_autocorr | ( |  | ) |  |

Gets the autocorrelation vector.

Returns
:   An std::unique\_ptr<std::vector<double>> containing the autocorrelation vector.

The elements of the vector are the index averaged mean values of <a\_i\*a\_i>, <a\_(i+1)\*a\_i>, <a\_(i+2)\*a\_i> ... Where a\_i are the pushed inside the buffer.

## ◆ push()

|  |  |  |  |  |  |  |  |
| --- | --- | --- | --- | --- | --- | --- | --- |
| |  |  |  |  |  |  | | --- | --- | --- | --- | --- | --- | | void autocorr::push | ( | const double & | *value* | ) |  | | virtual |

Pushes an element.

Parameters
:   |  |  |
    | --- | --- |
    | value | The pushed element. |

The pushed element will be available at object[get\_eff\_size()-1].

Reimplemented from buffer< double >.

---

The documentation for this class was generated from the following files:

- include/autocorr.h
- src/autocorr.cpp


---

Generated by  

 1.8.13
