# Supplementary material for: Spin-relaxation time in materials with broken inversion symmetry and large spin-orbit coupling
Source: Sci Rep. 2017 Aug 30;7:9949. doi: 10.1038/s41598-017-09759-0 (PMC5577210; doi:10.1038/s41598-017-09759-0)
Supplement: Supplementary file 2 — The Monte Carlo code of the calculations in C++ [file 41598_2017_9759_MOESM2_ESM.zip › DP_Monte_Carlo/doc/html/classSingleSpin-members.html]

Dyakonov Perel Monte Carlo simulation: Member List


|  |
| --- |
| Dyakonov Perel Monte Carlo simulation |

SingleSpin Member List

This is the complete list of members for SingleSpin, including all inherited members.

|  |  |  |
| --- | --- | --- |
| **B\_meas** (defined in SingleSpin) | SingleSpin | protected |
| B\_shot enum value | SingleSpin |  |
| **binary\_search\_t**(const double &t) (defined in SingleSpin) | SingleSpin | protected |
| burkov\_2d enum value | SingleSpin |  |
| burkov\_2d\_angle enum value | SingleSpin |  |
| burkov\_2d\_angle\_sx enum value | SingleSpin |  |
| burkov\_2d\_Sx enum value | SingleSpin |  |
| **delta\_omega** (defined in SingleSpin) | SingleSpin | protected |
| dresselhaus enum value | SingleSpin |  |
| dresselhaus\_xy enum value | SingleSpin |  |
| FillSzVec(std::vector< double > &Sz, const int &size, const double &dt) | SingleSpin |  |
| GetFirstTime() | SingleSpin | inline |
| GetLastTime() | SingleSpin | inline |
| GetSpin(const double &t) | SingleSpin |  |
| **kvecs** (defined in SingleSpin) | SingleSpin | protected |
| **meas** (defined in SingleSpin) | SingleSpin | protected |
| meas\_t enum name | SingleSpin |  |
| mixed\_3d enum value | SingleSpin |  |
| mn\_1d enum value | SingleSpin |  |
| **model** (defined in SingleSpin) | SingleSpin | protected |
| model\_t enum name | SingleSpin |  |
| naiv enum value | SingleSpin |  |
| **omega** (defined in SingleSpin) | SingleSpin | protected |
| prep enum value | SingleSpin |  |
| Print(std::ostream &out=std::cout) | SingleSpin |  |
| rashba\_3d enum value | SingleSpin |  |
| rashba\_dressel\_2d\_x enum value | SingleSpin |  |
| rashba\_dressel\_2d\_xy enum value | SingleSpin |  |
| rashba\_dressel\_2d\_z enum value | SingleSpin |  |
| rashba\_dressel\_3d\_111\_xx enum value | SingleSpin |  |
| rashba\_dressel\_3d\_111\_zz enum value | SingleSpin |  |
| rashba\_dressel\_3d\_x enum value | SingleSpin |  |
| rashba\_dressel\_3d\_xy enum value | SingleSpin |  |
| rashba\_dressel\_3d\_xz enum value | SingleSpin |  |
| rashba\_dressel\_3d\_z enum value | SingleSpin |  |
| RawPrint(std::ostream &out=std::cout) | SingleSpin |  |
| SingleSpin(const double &o=0.2, const double &deltao=0., const model\_t &m=naiv, const meas\_t &meas=prep, double B\_meas=0., double tmin=0.) | SingleSpin |  |
| **spins** (defined in SingleSpin) | SingleSpin | protected |
| Step() | SingleSpin | virtual |
| **times** (defined in SingleSpin) | SingleSpin | protected |
| **tmin** (defined in SingleSpin) | SingleSpin | protected |


---

Generated by  

 1.8.13
