# Supplementary material for: Spin-relaxation time in materials with broken inversion symmetry and large spin-orbit coupling
Source: Sci Rep. 2017 Aug 30;7:9949. doi: 10.1038/s41598-017-09759-0 (PMC5577210; doi:10.1038/s41598-017-09759-0)
Supplement: Supplementary file 2 — The Monte Carlo code of the calculations in C++ [file 41598_2017_9759_MOESM2_ESM.zip › DP_Monte_Carlo/doc/html/la_8h.html]

Dyakonov Perel Monte Carlo simulation: include/la.h File Reference


|  |
| --- |
| Dyakonov Perel Monte Carlo simulation |


- include

Functions

la.h File Reference

`#include <armadillo>`

Include dependency graph for la.h:

<p><b>This browser is not able to show SVG: try Firefox, Chrome, Safari, or Opera instead.</b></p>

Go to the source code of this file.

|  |  |
| --- | --- |
| Functions | |
| arma::vec | la::Rotate (const arma::vec &v0, const arma::vec &phi) |
|  | Rotates a vector around an other vecor. More... |
|  | |

## Function Documentation

## ◆ Rotate()

|  |  |  |  |
| --- | --- | --- | --- |
| arma::vec la::Rotate | ( | const arma::vec & | *v0*, |
|  |  | const arma::vec & | *phi* |
|  | ) |  |  |

Rotates a vector around an other vecor.

Parameters
:   |  |  |
    | --- | --- |
    | v0 | The vector to be rotated. |
    | phi | The angle vector. The length of this vector is the angle of the rotation in radians. The direction of the vector denotes the axis of the rotation. The rotation follows the right hand rule. |

Returns
:   The rotated vector.


---

Generated by  

 1.8.13
