# Supplementary material for: Spin-relaxation time in materials with broken inversion symmetry and large spin-orbit coupling
Source: Sci Rep. 2017 Aug 30;7:9949. doi: 10.1038/s41598-017-09759-0 (PMC5577210; doi:10.1038/s41598-017-09759-0)
Supplement: Supplementary file 2 — The Monte Carlo code of the calculations in C++ [file 41598_2017_9759_MOESM2_ESM.zip › DP_Monte_Carlo/doc/html/functions_enum.html]

Dyakonov Perel Monte Carlo simulation: Class Members - Enumerations


|  |
| --- |
| Dyakonov Perel Monte Carlo simulation |


- meas\_t
  : SingleSpin
- model\_t
  : SingleSpin


---

Generated by  

 1.8.13
