# Supplementary material for: Spin-relaxation time in materials with broken inversion symmetry and large spin-orbit coupling
Source: Sci Rep. 2017 Aug 30;7:9949. doi: 10.1038/s41598-017-09759-0 (PMC5577210; doi:10.1038/s41598-017-09759-0)
Supplement: Supplementary file 2 — The Monte Carlo code of the calculations in C++ [file 41598_2017_9759_MOESM2_ESM.zip › DP_Monte_Carlo/doc/html/classSingleSpin.html]

Dyakonov Perel Monte Carlo simulation: SingleSpin Class Reference


Dyakonov Perel Monte Carlo simulation

Public Types |
Public Member Functions |
Protected Member Functions |
Protected Attributes |
List of all members

SingleSpin Class Reference

Spin relaxation experiment class mainly for ensamble measurements.
More...

`#include <singlespin.h>`

Inheritance diagram for SingleSpin:

<p><b>This browser is not able to show SVG: try Firefox, Chrome, Safari, or Opera instead.</b></p>

[legend]

|  |  |
| --- | --- |
| Public Types | |
| enum | model\_t {     naiv, burkov\_2d, burkov\_2d\_Sx, burkov\_2d\_angle,     burkov\_2d\_angle\_sx, rashba\_3d, mixed\_3d, mn\_1d,     dresselhaus, dresselhaus\_xy, rashba\_dressel\_2d\_z, rashba\_dressel\_2d\_x,     rashba\_dressel\_2d\_xy, rashba\_dressel\_3d\_x, rashba\_dressel\_3d\_z, rashba\_dressel\_3d\_xz,     rashba\_dressel\_3d\_xy, rashba\_dressel\_3d\_111\_xx, rashba\_dressel\_3d\_111\_zz   }\|  \| An enum type for the underlying Hamiltonian and direction of interest. More... \| |
|  | |
| enum | meas\_t { prep, B\_shot }\|  \| Measurement type. More... \| |
|  | |

|  |  |
| --- | --- |
| Public Member Functions | |
|  | SingleSpin (const double &o=0.2, const double &deltao=0., const model\_t &m=naiv, const meas\_t &meas=prep, double B\_meas=0., double tmin=0.) |
|  | Constructor. More... |
|  | |
| virtual void | Step () |
|  | Advances the simulation. More... |
|  | |
| void | Print (std::ostream &out=std::cout) |
|  | Prints the electron states at the scattering events. |
|  | |
| void | RawPrint (std::ostream &out=std::cout) |
|  | Prints the electron states at the scattering events without pretty formatting. |
|  | |
| arma::vec | GetSpin (const double &t) |
|  | Gets the electron spin state at an arbitrary time within the simulation range. More... |
|  | |
| void | FillSzVec (std::vector< double > &Sz, const int &size, const double &dt) |
|  | Fills a vector with a given spin component at uniform time samples. More... |
|  | |
| double | GetFirstTime () |
|  | Gets the starting time of the simulation. More... |
|  | |
| double | GetLastTime () |
|  | Gets the time of the last scattering event. More... |
|  | |

|  |  |
| --- | --- |
| Protected Member Functions | |
| int | **binary\_search\_t** (const double &t) |
|  | |

|  |  |
| --- | --- |
| Protected Attributes | |
| model\_t | **model** |
|  | |
| meas\_t | **meas** |
|  | |
| double | **tmin** |
|  | |
| arma::vec | **B\_meas** |
|  | |
| double | **omega** |
|  | |
| double | **delta\_omega** |
|  | |
| std::vector< double > | **times** |
|  | |
| std::vector< arma::vec > | **kvecs** |
|  | |
| std::vector< arma::vec > | **spins** |
|  | |

## Detailed Description

Spin relaxation experiment class mainly for ensamble measurements.

It represents an experiment on a single spin. For an ensamble one has to create multiple classes an average over the results.

## Member Enumeration Documentation

## ◆ meas\_t

enum SingleSpin::meas\_t

Measurement type.

| Enumerator | |
| --- | --- |
| prep | Measurement starting from fully polarized spin state |
| B\_shot | Measurement starting from random spin state, external magnetic field is turned on at t=0 |

## ◆ model\_t

enum SingleSpin::model\_t

An enum type for the underlying Hamiltonian and direction of interest.

Each Hamiltonian has different built in SOC field, so they have different time evolutions. The direction of interest is relevant for the FillSzVec method. For a more detailed description of the models look at the main page.

| Enumerator | |
| --- | --- |
| naiv | Fully isotropic 3D model. |
| burkov\_2d | 2DEG model with Rashba SOC, z axis relaxation. |
| burkov\_2d\_Sx | 2DEG model with Rashba SOC, x axis relaxationa. |
| burkov\_2d\_angle | 2DEG model with Rashba SOC, spins started polarized at an angle to the z axis, z component gathered. |
| burkov\_2d\_angle\_sx | 2DEG model with Rashba SOC, spins started polarized at an angle to the z axis, x component gathered. |
| rashba\_3d | 3D model with Rashba SOC. |
| mixed\_3d | 3D model with both isotropic and Rashba SOC. |
| mn\_1d | 1D model which exactly gives back the result of motional narrowing. |
| dresselhaus | 3D model with Dresselhaus SOC, z axis relaxation |
| dresselhaus\_xy | 3D model with Dresselhaus SOC, spins started with x direction polarization, y component gathered. |
| rashba\_dressel\_2d\_z | 2DEG model with both rashba and Dresselhaus SOC, z axis relaxation. |
| rashba\_dressel\_2d\_x | 2DEG model with both rashba and Dresselhaus SOC, x axis relaxation. |
| rashba\_dressel\_2d\_xy | 2DEG model with both rashba and Dresselhaus SOC, spins started with x direction polarization, y component gathered. |
| rashba\_dressel\_3d\_x | 3D model with both Dresselhaus and Rashba SOC, x axis relaxation. |
| rashba\_dressel\_3d\_z | 3D model with both Dresselhaus and Rashba SOC, z axis relaxation. |
| rashba\_dressel\_3d\_xz | 3D model with both Dresselhaus and Rashba SOC, spins started with x direction polarization, z component gathered. |
| rashba\_dressel\_3d\_xy | 3D model with both Dresselhaus and Rashba SOC, spins started with y direction polarization, y component gathered. |
| rashba\_dressel\_3d\_111\_xx | 3D model with both Dresselhaus and Rashba SOC, [1,1,1] growth direction, x axis relaxation. |
| rashba\_dressel\_3d\_111\_zz | 3D model with both Dresselhaus and Rashba SOC, [1,1,1] growth direction, z axis relaxation. |

## Constructor & Destructor Documentation

## ◆ SingleSpin()

|  |  |  |  |
| --- | --- | --- | --- |
| SingleSpin::SingleSpin | ( | const double & | *o* = `0.2`, |
|  |  | const double & | *deltao* = `0.`, |
|  |  | const model\_t & | *m* = `naiv`, |
|  |  | const meas\_t & | *meas* = `prep`, |
|  |  | double | *B\_meas* = `0.`, |
|  |  | double | *tmin* = `0.` |
|  | ) |  |  |

Constructor.

Parameters
:   |  |  |
    | --- | --- |
    | o | The primary parameter for the SOC. It's typically the amplitude of the Larmor angular frequency due to the SOC field. The exact meaning depends on the model in question. |
    | deltao | The secondary paramter for the SOC. It typically describes the deviation of amplitude of the Larmor angular frequency. The exact meaning is model dependent. |
    | m | The model that selects the Hamiltonian, initial condition and the direction of interest. |
    | meas | The measurement type. |
    | B\_meas | The amplitude of the external magnetic field for B\_shot measurements. |
    | tmin | The starting time of the measurement. The magnetic field is turned on at t=0 for B\_shot measurements. |

## Member Function Documentation

## ◆ FillSzVec()

|  |  |  |  |
| --- | --- | --- | --- |
| void SingleSpin::FillSzVec | ( | std::vector< double > & | *Sz*, |
|  |  | const int & | *size*, |
|  |  | const double & | *dt* |
|  | ) |  |  |

Fills a vector with a given spin component at uniform time samples.

Parameters
:   |  |  |
    | --- | --- |
    | Sz | The vector to be filled. |
    | size | The number of points gathered. |
    | dt | The timestep between samples. |

## ◆ GetFirstTime()

|  |  |  |  |  |
| --- | --- | --- | --- | --- |
| double SingleSpin::GetFirstTime | ( |  | ) |  |

inline

Gets the starting time of the simulation.

Returns
:   The starting time of the simulation.

## ◆ GetLastTime()

|  |  |  |  |  |
| --- | --- | --- | --- | --- |
| double SingleSpin::GetLastTime | ( |  | ) |  |

inline

Gets the time of the last scattering event.

Returns
:   The time of the last scattering event.

## ◆ GetSpin()

|  |  |  |  |  |  |
| --- | --- | --- | --- | --- | --- |
| arma::vec SingleSpin::GetSpin | ( | const double & | *t* | ) |  |

Gets the electron spin state at an arbitrary time within the simulation range.

Returns
:   The electron spin state.

Exceptions
:   |  |  |
    | --- | --- |
    | std::runtime\_error | if t is not in range. |

## ◆ Step()

|  |  |  |  |  |
| --- | --- | --- | --- | --- |
| void SingleSpin::Step | ( |  | ) |  |

virtual

Advances the simulation.

The simulation is event driven. Each step advances the simulation to the next scattering event.

Reimplemented in SingleSpinAutocorr.

---

The documentation for this class was generated from the following files:

- include/singlespin.h
- src/singlespin.cpp


---

Generated by  

 1.8.13
