# Supplementary material for: Spin-relaxation time in materials with broken inversion symmetry and large spin-orbit coupling
Source: Sci Rep. 2017 Aug 30;7:9949. doi: 10.1038/s41598-017-09759-0 (PMC5577210; doi:10.1038/s41598-017-09759-0)
Supplement: Supplementary file 2 — The Monte Carlo code of the calculations in C++ [file 41598_2017_9759_MOESM2_ESM.zip › DP_Monte_Carlo/doc/html/graph_legend.html]

Dyakonov Perel Monte Carlo simulation: Graph Legend


Dyakonov Perel Monte Carlo simulation


Graph Legend

This page explains how to interpret the graphs that are generated by doxygen.

Consider the following example:

/\*! Invisible class because of truncation \*/

class Invisible { };

/\*! Truncated class, inheritance relation is hidden \*/

class Truncated : public Invisible { };

/\* Class not documented with doxygen comments \*/

class Undocumented { };

/\*! Class that is inherited using public inheritance \*/

class PublicBase : public Truncated { };

/\*! A template class \*/

template<class T> class Templ { };

/\*! Class that is inherited using protected inheritance \*/

class ProtectedBase { };

/\*! Class that is inherited using private inheritance \*/

class PrivateBase { };

/\*! Class that is used by the Inherited class \*/

class Used { };

/\*! Super class that inherits a number of other classes \*/

class Inherited : public PublicBase,

protected ProtectedBase,

private PrivateBase,

public Undocumented,

public Templ<int>

{

private:

Used \*m\_usedClass;

};

This will result in the following graph:

<p><b>This browser is not able to show SVG: try Firefox, Chrome, Safari, or Opera instead.</b></p> 

The boxes in the above graph have the following meaning:

- A filled gray box represents the struct or class for which the graph is generated.
- A box with a black border denotes a documented struct or class.
- A box with a gray border denotes an undocumented struct or class.
- A box with a red border denotes a documented struct or class forwhich not all inheritance/containment relations are shown. A graph is truncated if it does not fit within the specified boundaries.

The arrows have the following meaning:

- A dark blue arrow is used to visualize a public inheritance relation between two classes.
- A dark green arrow is used for protected inheritance.
- A dark red arrow is used for private inheritance.
- A purple dashed arrow is used if a class is contained or used by another class. The arrow is labelled with the variable(s) through which the pointed class or struct is accessible.
- A yellow dashed arrow denotes a relation between a template instance and the template class it was instantiated from. The arrow is labelled with the template parameters of the instance.


---

Generated by  

 1.8.13
