# Supplementary material for: Spin-relaxation time in materials with broken inversion symmetry and large spin-orbit coupling
Source: Sci Rep. 2017 Aug 30;7:9949. doi: 10.1038/s41598-017-09759-0 (PMC5577210; doi:10.1038/s41598-017-09759-0)
Supplement: Supplementary file 2 — The Monte Carlo code of the calculations in C++ [file 41598_2017_9759_MOESM2_ESM.zip › DP_Monte_Carlo/doc/html/functions_eval.html]

Dyakonov Perel Monte Carlo simulation: Class Members - Enumerator


Dyakonov Perel Monte Carlo simulation


- B\_shot
  : SingleSpin
- burkov\_2d
  : SingleSpin
- burkov\_2d\_angle
  : SingleSpin
- burkov\_2d\_angle\_sx
  : SingleSpin
- burkov\_2d\_Sx
  : SingleSpin
- dresselhaus
  : SingleSpin
- dresselhaus\_xy
  : SingleSpin
- mixed\_3d
  : SingleSpin
- mn\_1d
  : SingleSpin
- naiv
  : SingleSpin
- prep
  : SingleSpin
- rashba\_3d
  : SingleSpin
- rashba\_dressel\_2d\_x
  : SingleSpin
- rashba\_dressel\_2d\_xy
  : SingleSpin
- rashba\_dressel\_2d\_z
  : SingleSpin
- rashba\_dressel\_3d\_111\_xx
  : SingleSpin
- rashba\_dressel\_3d\_111\_zz
  : SingleSpin
- rashba\_dressel\_3d\_x
  : SingleSpin
- rashba\_dressel\_3d\_xy
  : SingleSpin
- rashba\_dressel\_3d\_xz
  : SingleSpin
- rashba\_dressel\_3d\_z
  : SingleSpin


---

Generated by  

 1.8.13
