# Supplementary material for: Spin-relaxation time in materials with broken inversion symmetry and large spin-orbit coupling
Source: Sci Rep. 2017 Aug 30;7:9949. doi: 10.1038/s41598-017-09759-0 (PMC5577210; doi:10.1038/s41598-017-09759-0)
Supplement: Supplementary file 2 — The Monte Carlo code of the calculations in C++ [file 41598_2017_9759_MOESM2_ESM.zip › DP_Monte_Carlo/doc/html/la_8h_source.html]

Dyakonov Perel Monte Carlo simulation: include/la.h Source File


Dyakonov Perel Monte Carlo simulation


- include

la.h

Go to the documentation of this file.

1 #ifndef LA\_H

2 #define LA\_H

3

5 #include <armadillo>

6

7 namespace la

8 {

18  arma::vec Rotate(const arma::vec& v0,const arma::vec& phi);

19 }

20

21 #endif

la

**Definition:** la.h:7

la::Rotate

arma::vec Rotate(const arma::vec &v0, const arma::vec &phi)

Rotates a vector around an other vecor.


---

Generated by  

 1.8.13
