# Supplementary material for: Spin-relaxation time in materials with broken inversion symmetry and large spin-orbit coupling
Source: Sci Rep. 2017 Aug 30;7:9949. doi: 10.1038/s41598-017-09759-0 (PMC5577210; doi:10.1038/s41598-017-09759-0)
Supplement: Supplementary file 2 — The Monte Carlo code of the calculations in C++ [file 41598_2017_9759_MOESM2_ESM.zip › DP_Monte_Carlo/doc/html/structProgress.html]

Dyakonov Perel Monte Carlo simulation: Progress Struct Reference


Dyakonov Perel Monte Carlo simulation

Public Member Functions |
Public Attributes |
List of all members

Progress Struct Reference

|  |  |
| --- | --- |
| Public Member Functions | |
| void | **MLSend** (MLINK lp) |
|  | |

|  |  |
| --- | --- |
| Public Attributes | |
| std::string | **Mathematica\_variable** |
|  | |
| double | **progress** |
|  | |

---

The documentation for this struct was generated from the following file:

- mathlink/mathlink.cpp


---

Generated by  

 1.8.13
